# Identification of changes in dendritic cell subsets that correlate with disease severity in dengue infection

**DOI:** 10.1371/journal.pone.0200564

**Published:** 2018-07-12

**Authors:** Sakaorat Lertjuthaporn, Ladawan Khowawisetsut, Rassamon Keawvichit, Korakot Polsrila, Ampaiwan Chuansumrit, Kulkanya Chokephaibulkit, Premrutai Thitilertdecha, Nattawat Onlamoon, Aftab A. Ansari, Kovit Pattanapanyasat

**Affiliations:** 1 Graduate Program in Immunology, Department of Immunology, Faculty of Medicine Siriraj Hospital, Mahidol University, Bangkok, Thailand; 2 Department of Parasitology, Faculty of Medicine Siriraj Hospital, Mahidol University, Bangkok, Thailand; 3 Department of Research and Development, Faculty of Medicine Siriraj Hospital, Mahidol University, Bangkok, Thailand; 4 Department of Pediatrics, Faculty of Medicine Ramathibodi Hospital, Mahidol University, Bangkok, Thailand; 5 Department of Pediatrics, Faculty of Medicine Siriraj Hospital, Mahidol University, Bangkok, Thailand; 6 Research Group in Immunobiology and Therapeutic Sciences, Faculty of Medicine Siriraj Hospital, Mahidol University, Bangkok, Thailand; 7 Department of Pathology and Laboratory Medicine, Emory University School of Medicine, Atlanta, Georgia, United States of America; University of Hong Kong, HONG KONG

## Abstract

Dengue virus (DENV) is the most prevalent arthropod-borne viral disease in humans. DENV causes a spectrum of illness ranging from mild to potentially severe complications. Dendritic cells (DCs) play a critical role in initiating and regulating highly effective antiviral immune response that include linking innate and adaptive immune responses. This study was conducted to comparatively characterize in detail the relative proportion, phenotypic changes, and maturation profile of subsets of both myeloid DCs (mDCs) and plasmacytoid DCs (pDCs) in children with dengue fever (DF), dengue hemorrhagic fever (DHF) and for purposes of control healthy individuals. The mDCs (Lin^-^CD11c^+^CD123^lo^), the pDCs (Lin^-^CD11c^-^CD123^+^) and the double negative (DN) subset (Lin^-^/HLA^-^DR^+^/CD11c^-^CD123^-^) were analyzed by polychromatic flow cytometry. The data were first analyzed on blood samples collected from DENV-infected patients at various times post-infection. Results showed that the relative proportion of mDCs were significantly decreased which was associated with an increase in disease severity in samples from DENV-infected patients. While there was no significant difference in the relative proportion of pDCs between healthy and DENV-infected patients, there was a marked increase in the DN subset. Analysis of the kinetics of changes of pDCs showed that there was an increase but only during the early febrile phase. Additionally, samples from patients during acute disease showed marked decreases in the relative proportion of CD141^+^ and CD16^+^ mDC subsets that were the major mDC subsets in healthy individuals. In addition, there was a significant decrease in the level of CD33-expressing mDCs in DENV patients. While the pDCs showed an up-regulation of maturation profile during acute DENV infection, the mDCs showed an alteration of maturation status. This study suggests that different relative proportion and phenotypic changes as well as alteration of maturation profile of DC subsets may play a critical role in the dengue pathogenesis and disease outcome.

## Introduction

Dengue virus (DENV) is the most prevalent arthropod-borne viral disease in humans. The virus is transmitted to humans via infected female *Aedes aegypti* or *Aedes albopictus* mosquitoes. DENV is classified into four antigenically distinct serotypes, DENV-1, DENV-2, DENV-3, and DENV-4 [1]. Dengue disease is a global epidemic involving more than 100 countries predominantly within tropical and subtropical regions [2]. Furthermore, it is estimated that approximately 2.5 billion people are now at risk of DENV infection. One recent study indicated that there are 400 million dengue infections per year of which approximately 100 million resulted in symptomatic cases associated with 21,000 deaths [3]. Most DENV infections are asymptomatic, although DENV can cause a spectrum of illness ranging from mild symptoms denoted as dengue fever (DF) to potentially severe manifestations that include dengue hemorrhagic fever (DHF) and dengue shock syndrome (DSS). The 1997 WHO guideline, which has been used in this study, classifies DHF into 4 grades of severity, with grade III and IV being classified as DSS. The clinical manifestations of DHF are enhancement of vascular permeability, endothelial cell damage and plasma leakage [4] that are the predominant cause of death [5]. There is presently no licensed vaccines or effective antiviral drugs available. The question why do only some patients develop severe manifestations after DENV infection continues to be a subject of debate. There have been several proposed hypotheses describing the development of severe DENV manifestations. These include exacerbated T cell response with elevated level of cytokines (cytokine storms), as well as antibody-dependent enhancement (ADE). The latter implicates pre-existing sub-neutralizing antibodies induced by the first DENV infection that bind to DENV particles of the second infection with heterotypic serotypes [6–8].

Both innate and adaptive immune response have been characterized in patients with DENV infection. Among the cell lineages involved, the dendritic cells (DCs) are of particular interest because of their link between innate and adaptive immune responses and their critical role in the induction and regulation of potent anti-viral immune responses [9]. It is important to note that dermal DCs and Langerhan cells have been noted to be the primary targets of dengue virus infection following transmission [10]. Upon DENV infection, DCs undergo maturation, which is characterized by upregulated expression of major histocompatibility class II (MHC II) antigens and the co-stimulatory molecules CD40, CD80 (B7-1), CD83, and CD86 (B7-2) [11, 12]. DENV infection may induce DC apoptosis and decrease their ability to optimally prime and present antigen to CD4+ or CD8+ T-cells that potentially contribute to DENV pathogenesis [13]. In the peripheral blood, there are two major subsets of human blood DCs. These include the myeloid DCs (mDCs) and the plasmacytoid DCs (pDCs) [14, 15]. Both mDCs and pDCs are characterized by the expression of HLA-DR, and the absence of a number of cell lineage (lin) markers such as CD3, CD14, CD19 and CD56. The mDCs (lin^-^HLA-DR^+^CD11c^+^CD123^lo^) play a major role in antigen capture, processing and presentation to T cells. DCs are distributed in peripheral tissues and following antigen capture have the ability to migrate to lymphoid organs, where they interact with T cells to orchestrate immune responses. The mDCs are further subdivided into three subsets, the CD16^+^ mDCs, the CD1c^+^ mDCs and the CD141^+^ mDCs [14, 16, 17]. The CD16^+^ mDCs have been previously reported to have a relatively potent pro-inflammatory function associated with high production of TNF-α in response to most TLR agonists [16]. The CD1c^+^ DCs appear to be primarily involved in the presentation of lipid and glycolipid antigens to T cells [18]. Of interest is the finding that while the group 1 CD1 isoforms that include CD1a, CD1b and CD1c have structurally diverse antigen binding grooves, they have similar roles in antigen presentation to T cells. The fact that there are no known data on the CD1b^+^ mDC subset prompted us to focus on characterizing this subset in dengue infected patients. The CD141^+^ DC subset have been shown to promote Th1 type of immune responses following TLR3 stimulation in addition to be involved in necrotic cell antigen cross-presentation [19]. The pDCs (lin^-^HLA-DR^+^CD11c^-^CD123^+^) on the other hand are well recognized as a major source of type I interferon (IFN) that contributes to limiting viral replication [20].

There have been a number of previous studies that have described the frequencies of mDCs and pDCs in dengue patients. However, these previous studies were cross sectional and included description of total populations of mDCs and pDCs without addressing the role of subsets within these cell lineages. Thus, one of these studies documented reduced absolute numbers of both mDCs and pDCs in dengue patients as compared to healthy adults but these decreases did not correlate with disease severity but with levels of viremia [21]. A separate study documented decreases in total mDCs during acute viral illness and the decrease was correlated with disease severity [22]. In this study, there was also decreases in the levels of pDCs during acute viral illness but only in those patients that went onto develop DHF [22]. Finally, a more recent study documented an increase in the frequencies of mDCs associated with disease severity but this was an increase only in the cytokine expressing mDCs [23]. We reasoned that a more detailed study of the relative proportion of mDCs/pDCs, their subsets and levels of maturation that also included longitudinal studies on a subset of dengue infected patients may provide some important clues as to the potential roles of these cells in dengue pathogenesis. Hence the rationale for the studies reported herein.

## Materials and methods

### Sample collection and study population

DENV infected patients were enrolled from Department of Pediatrics at Ramathibodi Hospital and Siriraj Hospital, Mahidol University, Bangkok, Thailand. Siriraj Institutional Review Board, Faculty of Medicine Siriraj Hospital, Mahidol University approved the study (Si 092/2010) and written informed consent was obtained from all subjects. Sixty-eight blood samples from the twenty-two DENV-infected patients were drawn using sodium citrate-containing tubes daily after enrollment until hospital discharge. Healthy age-matched volunteer subjects ages 3–13 (n = 14) were enrolled as controls in this study. It should be noted that blood samples were collected at different days of fever ranging from day -2 (D-2) to day +2 (D+2). Thus, samples collected on D-2 to D-1 were considered as the febrile phase, those collected during D0, day of defervescence when the temperature dropped below 37.5°C without subsequently elevation to D+1 representing the defervescence phase, and those collected during D+2 representing the recovery phase or afebrile phase.

Patients were diagnosed by physicians using clinical presentations and routine laboratory tests including complete blood count, urinalysis, and blood chemistry. Subjects suspected of DENV infection were confirmed using reverse transcription-polymerase chain reaction (RT-PCR) and serology using the Platelia^TM^ Dengue NS1 Ag microplate EIA (Hercules, CA). DENV-infected patients were characterized according to the 1997 WHO guidelines and were classified as DF (n = 12) and DHF (n = 10) grades 1–3. Demographic characteristics of the study population are shown in Table 1.

**Table 1:** Demographic characteristics of the study population.

Subjects	Number of subjects	Gender (male:female)	Mean age, years (range)	DENV serotypes			
				DENV1	DENV2	DENV3	DENV4
DF	12	6:6	13 (10–18)	1	2	3	6
DHF (grade I, II, III)	10 (5, 3, 2)	5:5	12 (7–15)	2	1	3	4
Healthy individuals	14	8:6	10 (3–13)				
Total				3	3	6	10

DENV, dengue virus; DF, dengue fever; DHF, dengue hemorrhagic fever; grade, levels of severity followed 1997 WHO criteria.

### Identification of dengue virus serotype

Plasma was collected from the blood sample. Total RNA was extracted from patient’s plasma sample by using QIAamp viral RNA extraction kit (Qiagen, Germany) according to the manufacturer’s instruction. The detection of DENV serotypes (DENV 1–4) was determined by a multiplex nested RT-PCR. Briefly, the target viral RNA was reverse transcribed to cDNA by reverse transcriptase prior to PCR amplification using a pair of Env (E) primers. The PCR product was subjected to a multiplex nested PCR using a set of four primer pairs specific for sequences within the Env region of four DENV serotypes (S1 Table).

### Monoclonal antibodies used for flow cytometry

The fluorochrome-conjugated anti-human monoclonal antibodies (mAbs) used for cell surface staining included fluorescein isothiocyanate (FITC)-conjugated mAb to CD3 (clone SK7, T cell marker) and CD33 (clone HIM3-4, Siglec-3), phycoerythrin (PE)-conjugated mAb to HLA-DR (clone L243, MHC class II cell surface receptor), PE-Texas Red conjugated mAb to CD19 (clone SJ25-C1, B cell marker), PE/Dazzle594 conjugated mAb to CD86 (clone IT2.2, maturation marker of DC), peridinin chlorophyll protein (PerCP)-conjugated mAb to CD45 (clone 2D1, pan-human leukocyte antigen), phycoerythrin/cyanine7 (PE-Cy7)-conjugated mAb to CD304 (clone 12C2, pDC marker) and CD83 (clone HB15e, maturation marker of DC), allophycocyanine (APC)-conjugated mAb-CD7 (clone CD7-6B7, NK cell marker) and CD1b (clone 737249, mDC subset marker), Alexa Fluor® 700 (A700)-conjugated mAb to CD11c (clone Bu15, mDC marker), allophycocyanin/ cyanine 7 (APC-Cy7)-conjugated mAb to CD3 (clone SK7), CD19 (clone HIB19) and CD56 (clone HCD56) (T cell, B cell, and NK cell markers, respectively), Pacific Blue-conjugated mAb to CD40 (clone 5C3, maturation marker of DC), BV421-conjugated mAb to CD141 (clone M80, mDC subset marker), BV510-conjugated mAb to CD16 (clone 3G8, FcγIII receptor), BV570-conjugated mAb to CD14 (clone M5E2, monocyte marker), BV605-conjugated mAb to CD80 (clone 2D10, maturation marker of DC), and BV650-conjugated mAb to CD123 (clone 6H6, pDC marker). The controls for the staining protocol including those for the maturation markers were similar isotype control antibodies conjugated to FITC, Pacific Blue, PE-Texas Red, PE-Cy7 and BV605 were applied. Most mAbs were purchased from Biolegend (San Diego, CA), except CD3-FITC and CD45-PerCP from BD Biosciences (San Jose, CA), CD19-PE-Texas Red from SouthernBiotech (Birmingham, USA) and CD1b-APC from R&D systems (Minneapolis, MN).

### Immunofluorescent staining

Three staining panels of mAbs for characterization of pDCs, mDC, and its subsets and stage of maturation of DCs are shown in S2 Table. Two hundred microliters from each whole blood samples were incubated with appropriate mAbs in a 12×75-mm polystyrene tube for 15 minutes at room temperature in the dark. After incubation, 2 ml of 1X FACSlysing solution (BD Biosciences) was added, mixed and the cell suspension incubated for another 15 minutes to lyse the red blood cells. The stained sample was washed and centrifuged with 2 ml of phosphate buffered saline (PBS). Finally, the stained samples were re-suspended in 300 μl of PBS and kept at 4°C and subjected to polychromatic flow cytometric analysis.

### Flow cytometric analysis

**Fig 1:**
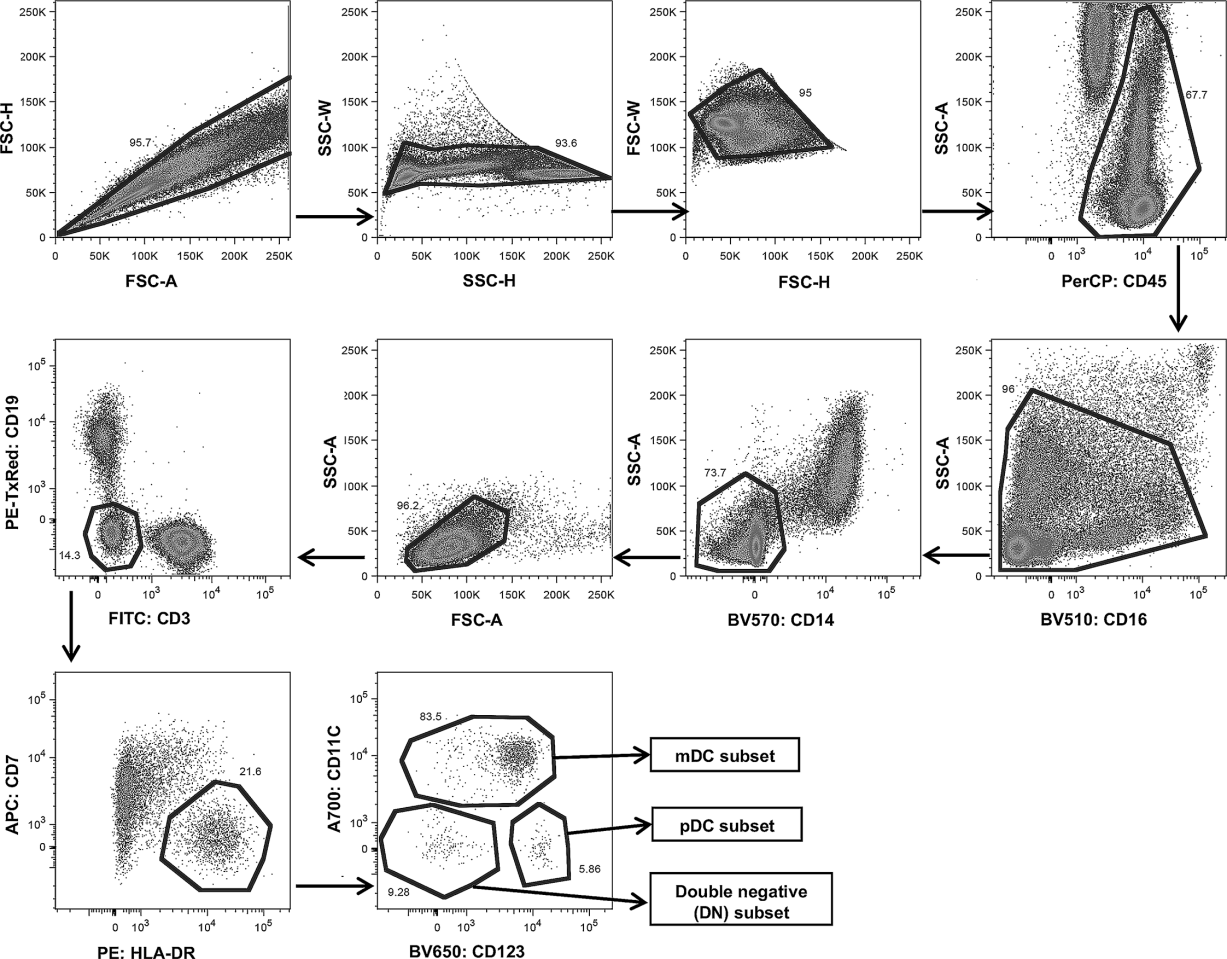
Gating strategy for the analysis of DCs from peripheral blood of DENV-infected patient. Two major populations of DC subsets were identified from CD45^+^ cells followed by CD16 of both large and small cells and those with different levels of granularity. The CD45^+^/CD14^-^/CD3^-^/CD19^-^/CD7^-^/HLA-DR^+^ cells were further analysed for mDCs as identified by their CD11c^+^CD123^lo^ and pDCs which were identified as CD11c^-^CD123^+^. The percentage of double negative (DN) subset (CD11c^-^CD123^-^) were also noted.

Data acquisition and analysis were performed on a LSRFortessa flow cytometer (BD Bioscience) using FACSDiva software (BD Bioscience). Post-acquisition analysis was performed using FlowJo software (Tree Star, Ashland, OR). The cell population from DENV-infected patient (Fig 1) and healthy individual (S1 Fig) were initially analyzed and followed by doublet discrimination, FSC-H/FSC-A, SSC-W/SSC-H, and FSC-W/FSC-H, respectively. Singlet cells were gated based on SSC-A/CD45 including lymphocytes and monocytes. DCs were gated as lineage negative (Lin^-^) cells by excluding CD16^bright^ (neutrophil and eosinophil), CD14^+^ (monocytes), CD3^+^ (T cells), CD19^+^ (B cells), and CD7^+^ (NK cells). HLA-DR^+^/Lin^-^ cells were further divided into three populations, mDCs (CD11c^+^CD123^lo^), pDCs (CD11c^-^CD123^+^), and a double negative (DN) subset (CD11c^-^CD123^-^). The mAbs against CD40, CD80, CD83, and CD86 on both mDCs and pDCs were used to define the stages of maturation on both mDCs and pDCs and the use of isotype controls for each utilized for fine analysis. Moreover, the expression of CD33 (Siglec-3), a transmembrane receptor primarily expressed by myeloid cells including both mDCs and pDCs was also analyzed. For mDC subset analysis, a different gating strategy was utilized. Thus, following doublet discrimination, mDCs were first gated on HLA-DR^+^/Lin^-^ cells followed by gated on cell population that express CD11c without CD304 and CD123 expression. The mDC subsets were subsequently identified as those that expressed CD16^+^, CD1b^+^ or CD141^+^ (S2 Fig). Flow cytometric data were expressed as relative proportion (%) and mean fluorescence intensity (MFI) of cells expressing each antigen. It should be noted that the relative proportion of these three subsets of mDCs were calculated as a percentage of the total number of mDCs.

### Statistical analysis

GraphPad Prism 6 statistical software was used for all statistical analyses. Since the data set for each study group was not normally distributed, either the Kruskal-Wallis test with Dunn’s multiple comparison post-tests or the Mann-Whitney U test was used to compare data more than two groups and between two groups. The numerical data were expressed as the medians and 25% to 75% interquartile ranges. The statistical significant threshold for all comparisons was set at p value less than 0.05.

## Results

### Decreased relative proportion of mDCs correlates with disease severity

**Fig 2:**
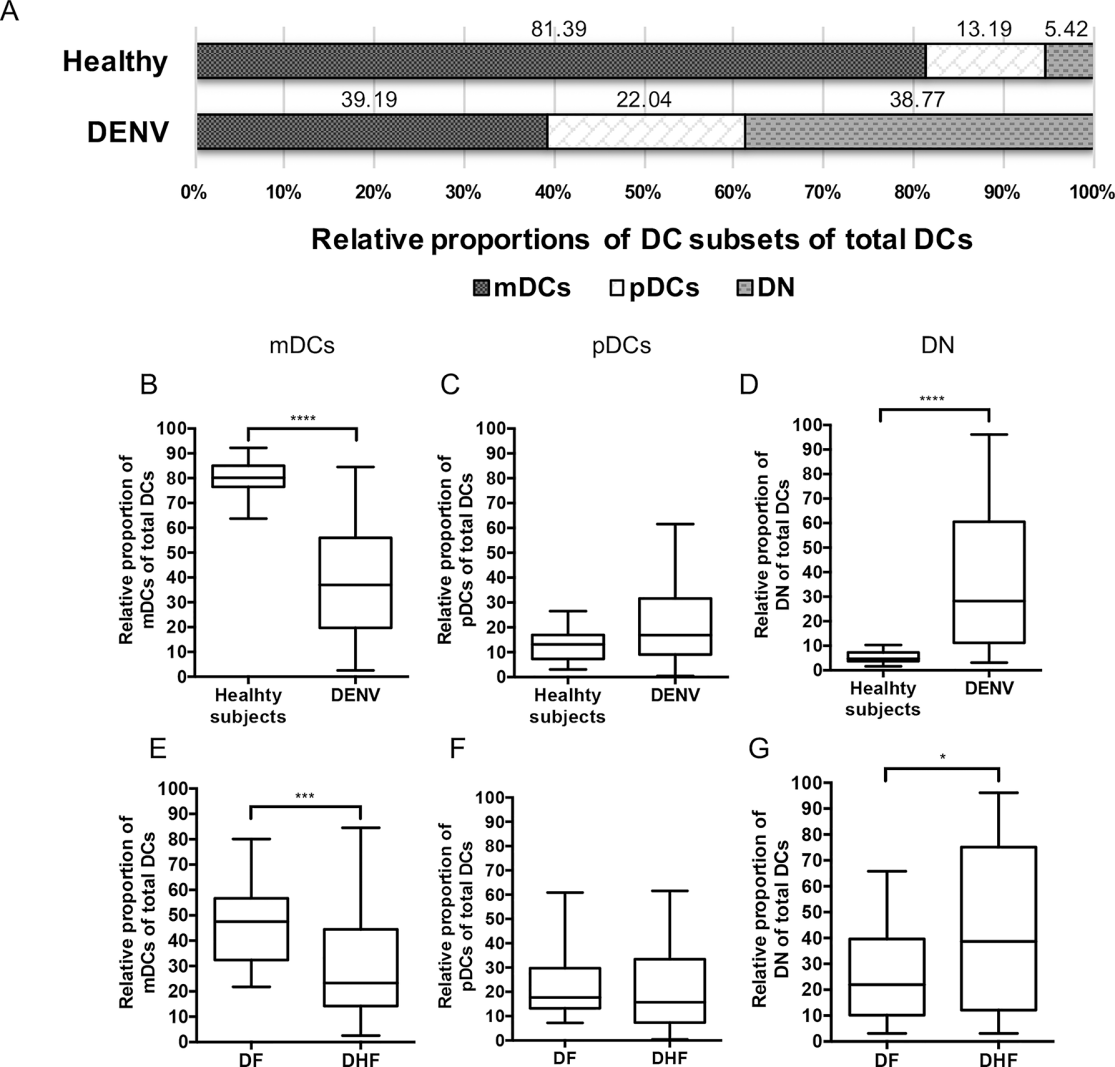
Comparison of the relative proportion of mDCs, pDCs and DN subset in blood samples from DENV infected patients and healthy subjects. The mean of relative proportion (%) of mDCs, pDCs, and DN were analyzed in blood samples from 68 DENV-infected samples and 14 healthy returned subjects (A). The relative proportion of mDCs (B) pDCs (C) and DN subsets (D) were compared between healthy and DENV-infected patients. The relative proportion of mDCs (E), pDCs (F) and DN subsets (G) were compared between DF and DHF patients. The box plot shows the median value (horizontal line in the box). The box and whisker represent 25^th^ to 75^th^, and 10^th^ to 90^th^ interquartile range, respectively. P values were determined by the Mann-Whitney U test for comparison of two groups. (* p < 0.05, *** p < 0.001 and **** p < 0.0001).

The relative proportion of mDCs, pDCs and the non-mDC/non-pDC-DN subset were first analyzed in blood samples collected from DENV-infected patients at various times during infection and compared with values obtained on blood samples from healthy subjects. The mean of relative proportion (%) of mDCs, pDCs and DN subset from peripheral blood of healthy subjects were 81.39%, 13.19% and 5.42%, respectively. In contrast, the mean of relative proportion (%) of mDCs, pDCs and DN subset from peripheral blood from the DENV-infected patients were 39.19%, 22.04% and 38.77%, respectively (Fig 2A). As seen, the relative proportion of mDCs in DENV-infected patients were significantly (p<0.0001) decreased when compared with that of healthy individuals (Fig 2B). In contrast, the relative proportion of DN subset in DENV-infected patients were significantly (p<0.0001) higher than that of healthy individuals (Fig 2D). However, there was no significant difference in the relative proportion of pDCs between healthy individuals and DENV-infected patients (Fig 2C).

In an effort to determine whether these differences were correlated with severity of disease, the data on the relative proportion of DCs during acute disease were compared between thirty-one samples of DF and thirty-seven samples of DHF (Fig 2E–2G). As seen in Fig 2E, the relative proportion of mDCs in DHF patients were significantly (p<0.001) lower than those in DF patients accompanied by significant increases (p = 0.03) in the relative proportion of DN subset in DHF patients as compared with DF patients (Fig 2G). There was no significant difference in the relative proportion of pDCs between DF and DHF patients (Fig 2F). These data suggest that a decrease in the relative proportion of mDCs but not pDCs is associated with the disease severity during acute DENV infection.

### Differences in subsets of mDC and CD33-expressing mDCs during acute DENV infection

**Fig 3:**
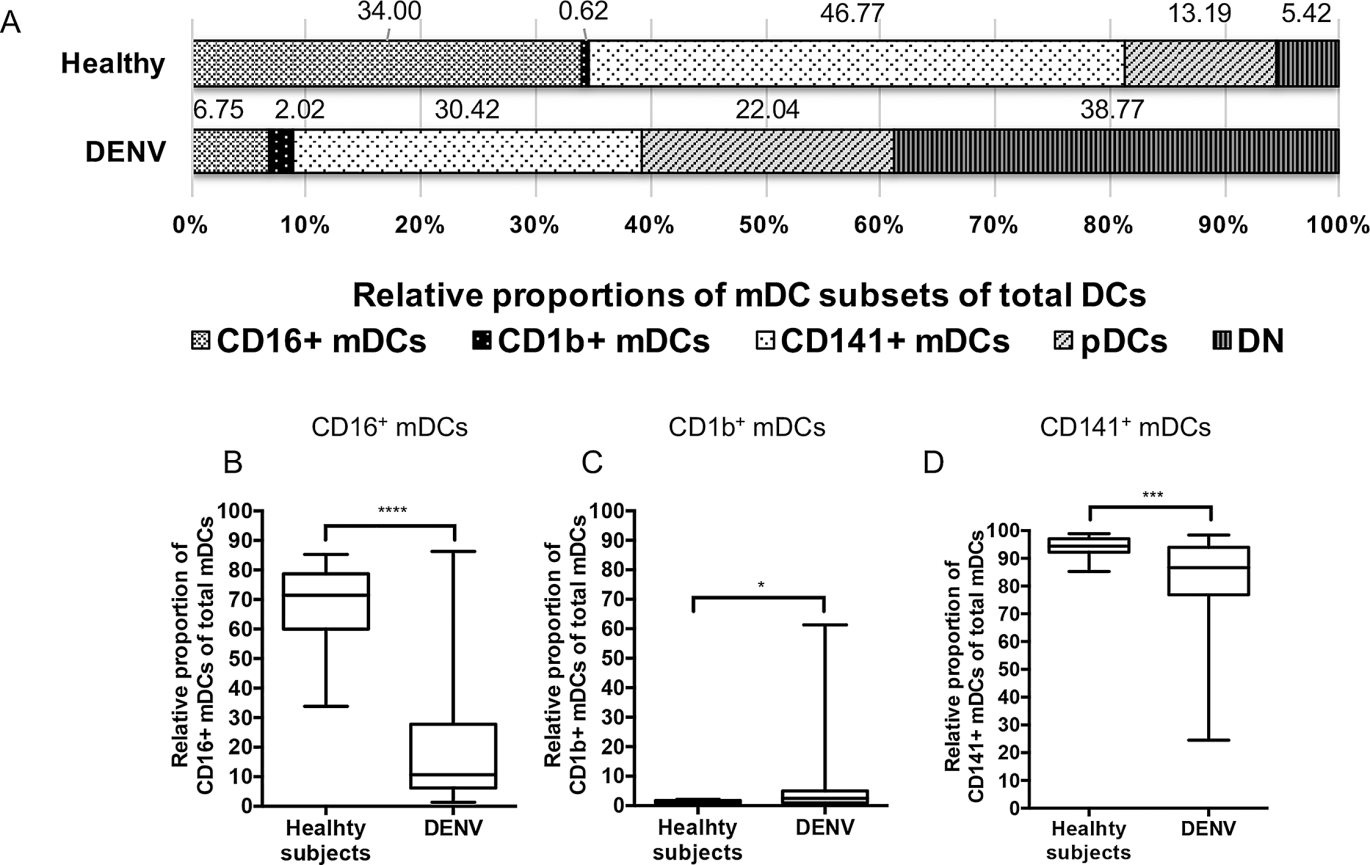
Comparison of the relative proportion of mDC subsets in DENV infected patients. Distribution of each of the three mDC subsets as a percentage of total DCs during acute DENV was analyzed from 64 DENV-infected samples and 14 healthy subjects (A). The relative proportion (%) of CD16^+^ mDCs (B), CD1b^+^ mDCs (C) and CD141^+^ mDCs (D) as a percentage of total mDCs in DENV-infected patients were compared with those of healthy subjects. The box plot shows the median value (horizontal line in the box). The box and whisker represent 25^th^ to 75^th^, and 10^th^ to 90^th^ interquartile range, respectively. P values were determined by the Mann-Whitney U test for comparison between two groups. (* p < 0.05, *** p <0.001 and **** p < 0.0001).

Since one of the major difference between dengue infected patients and normal subjects was noted in the relative proportion of mDCs, we reasoned that a more detailed analysis of the mDC subsets that contribute to such difference would be informative. We thus first investigated the relative proportion of mDCs that either expressed CD16, CD1b or CD141 (comprising the three distinct subsets of mDC) as a percentage of total DCs. The distribution of each of these mDC subset during acute DENV was analyzed from 64 DENV-infected samples and 14 healthy subjects. The mean of relative proportion (%) of CD16^+^, CD1b^+^ and CD141^+^ mDC subsets among total DCs from the peripheral blood of healthy subjects were 34.00%, 0.62% and 46.77%, respectively, while the mean of relative proportion (%) of CD16^+^, CD1b^+^ and CD141^+^ mDCs subsets among total DCs from peripheral blood of DENV-infected patients were 6.75%, 2.02% and 30.42%, respectively (Fig 3A). Secondly, we analyzed the relative proportion of the three mDC subsets as a percentage of total mDCs. As seen in Fig 3B and 3D, the relative proportion of CD16^+^ mDCs and CD141^+^ mDCs in DENV-infected patients were found to be significantly decreased when compared with those of healthy individuals with p<0.0001 and p<0.001, respectively. In contrast, the relative proportion of the CD1b^+^ mDCs subset in DENV-infected patients was significantly (p = 0.02) higher than that of healthy individuals (Fig 3C). There was no significant difference in the relative proportion of the three mDC subsets between DF and DHF patients (data not shown).

**Fig 4:**
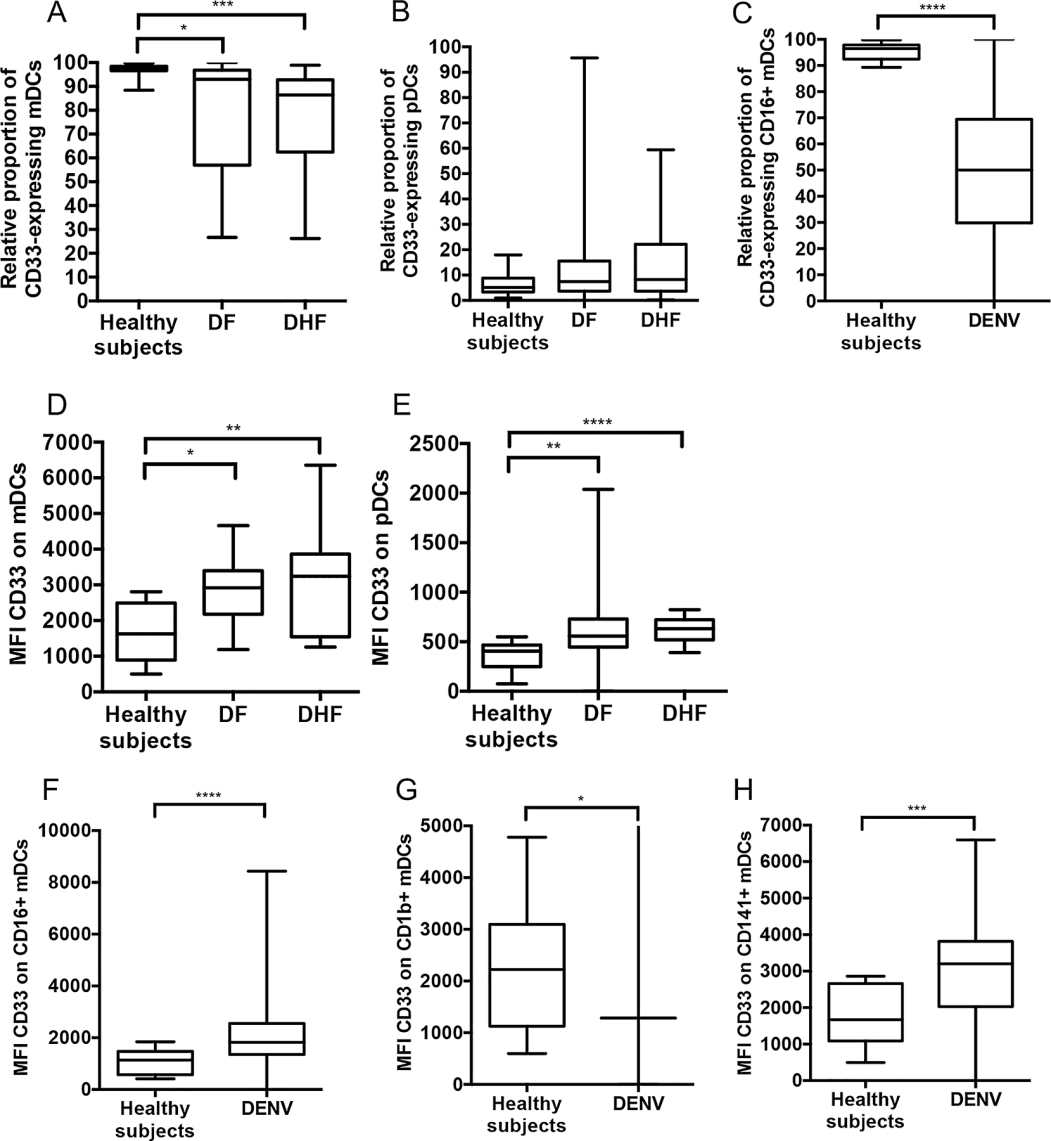
Comparison of the relative proportion and MFI of CD33-expressing mDCs and pDCs during acute DENV infection. The relative proportion of CD33-expressing mDCs (A) and pDCs (B) were compared between three groups, healthy subjects, DF and DHF patients. The relative proportion of CD33-expressing CD16^+^ mDCs in DENV-infected patients were compared with healthy individuals (C). The MFI of CD33 on mDCs (D), pDCs (E), CD16^+^ mDCs (F), CD1b^+^ mDCs (G), CD141^+^ mDCs (H) were compared between healthy subjects and DENV-infected patients. The box plot shows the median value (horizontal line in the box). The box and whisker represent 25^th^ to 75^th^, and 10^th^ to 90^th^ interquartile range, respectively. P values were determined by the Dunn’s post-test after Kruskal-Wallis test for comparison of three groups or Mann-Whitney U test for comparison between two groups. (* p < 0.05, ** p < 0.01, *** p < 0.001 and **** p < 0.0001).

DCs much like all myeloid cells express sialic acid-binding immunoglobulin-like lectins (Siglec) -3 or CD33, an immune inhibitory receptor. The removal of cell surface sialic acids by neuraminidase treatment triggers DC maturation by increasing the expression of MHC class I and II, CD80 and CD86 [24]. Thus, we also evaluated the expression level of CD33 on mDCs and pDCs in DENV-infected patients. The representative histogram plots of expression level of the CD33 on mDCs and pDCs were analyzed by polychromatic flow cytometry (S3 Fig). First of all, the relative proportion of CD33-expressing mDCs in both DF and DHF patients were significantly decreased when compared with those of healthy individuals with p = 0.015 and p<0.001, respectively (Fig 4A). However, there was no significant difference in the relative proportion of CD33-expressing pDCs (Fig 4B). Secondly, we examined the expression of CD33 on the three mDC subsets. As noted, while the relative proportion of CD33-expressing CD16^+^ mDCs in DENV-infected patients was significant lower than that of healthy individuals with p<0.0001 (Fig 4C), there was no significant difference in the relative proportion of CD33-expressing CD1b^+^ and CD141^+^ mDCs (S4 Fig). The MFI of CD33 expression on mDCs and pDCs in DENV-infected patients were significant higher than that of healthy individuals (Fig 4D and 4E). The MFI of CD33 expression on CD16^+^ and CD141^+^ mDCs in DENV-infected patients were significantly (p<0.001, p<0.0001, respectively) increased when compared with that of healthy individuals (Fig 4F and 4H). In contrast, The MFI of CD33 expression on CD1b^+^ mDCs in DENV-infected patients were significantly (p = 0.04) lower than that of healthy individuals (Fig 4G).

### Impact of acute DENV infection on mDCs and pDCs maturation

**Fig 5:**
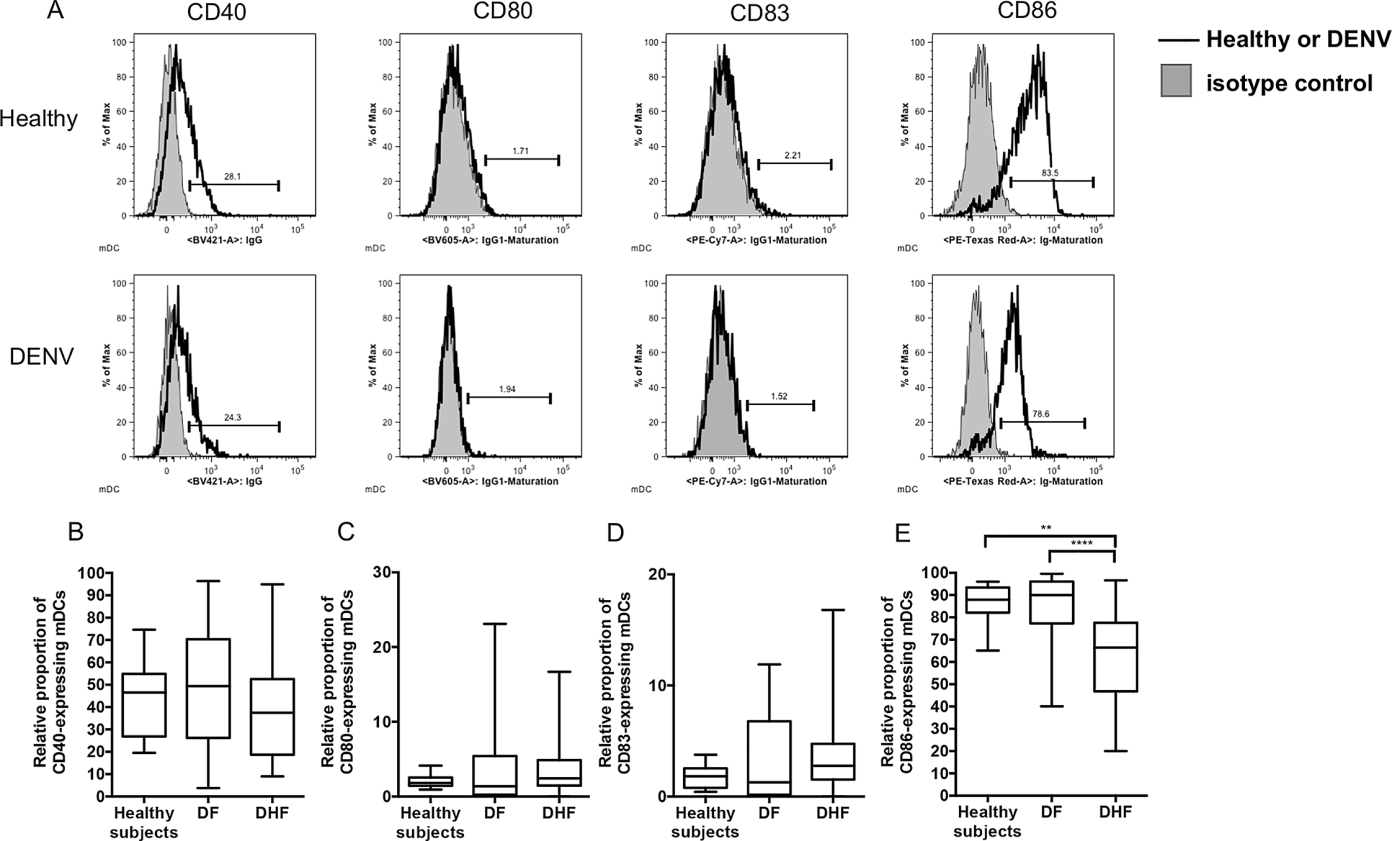
Comparison of the expression of potential markers of relative mDCs maturation during acute DENV infection. (A) Representative histogram plots showing maturation markers on mDCs on blood samples from healthy subjects (solid black line, upper panel) and DENV-infected patients (solid black line, lower panel). The shaded grey histograms represent profiles obtained using isotype controls. The relative proportion of CD40 (B), CD80 (C), CD83 (D), and CD86 (E) -expressing mDCs were compared on samples from healthy subjects and DENV-infected patients, DF and DHF patients. The box plot shows the median value (horizontal line in the box). The box and whisker represent 25^th^ to 75^th^, and 10^th^ to 90^th^ interquartile range, respectively. P values were determined by the Dunn’s post-test after Kruskal-Wallis test for comparison of three groups. (** p < 0.01 and **** p < 0.0001).

Findings of a significant decrease in the relative proportion of CD33-expressing mDCs prompted us to determine whether such a decrease resulted in changes in the maturation stages of mDCs. To evaluate the levels of maturation of mDCs in DENV-infected patients and healthy subjects, the representative histogram plots of expression levels of the co-stimulatory molecules, CD40, CD80, CD83, and CD86 were analyzed by polychromatic flow cytometry (Fig 5A). There was no significant difference in the relative proportion of CD40, CD80 and CD83 -expressing mDCs between DENV-infected patients and healthy individuals (Fig 5B–5D). The relative proportion of CD86-expressing mDCs in DHF patients was significantly lower than that of healthy individuals (p<0.0001) and DF patients (p<0.01) (Fig 5E). In contrast, there was no significant difference in the MFI of CD86-expressing mDCs (S5A Fig).

**Fig 6:**
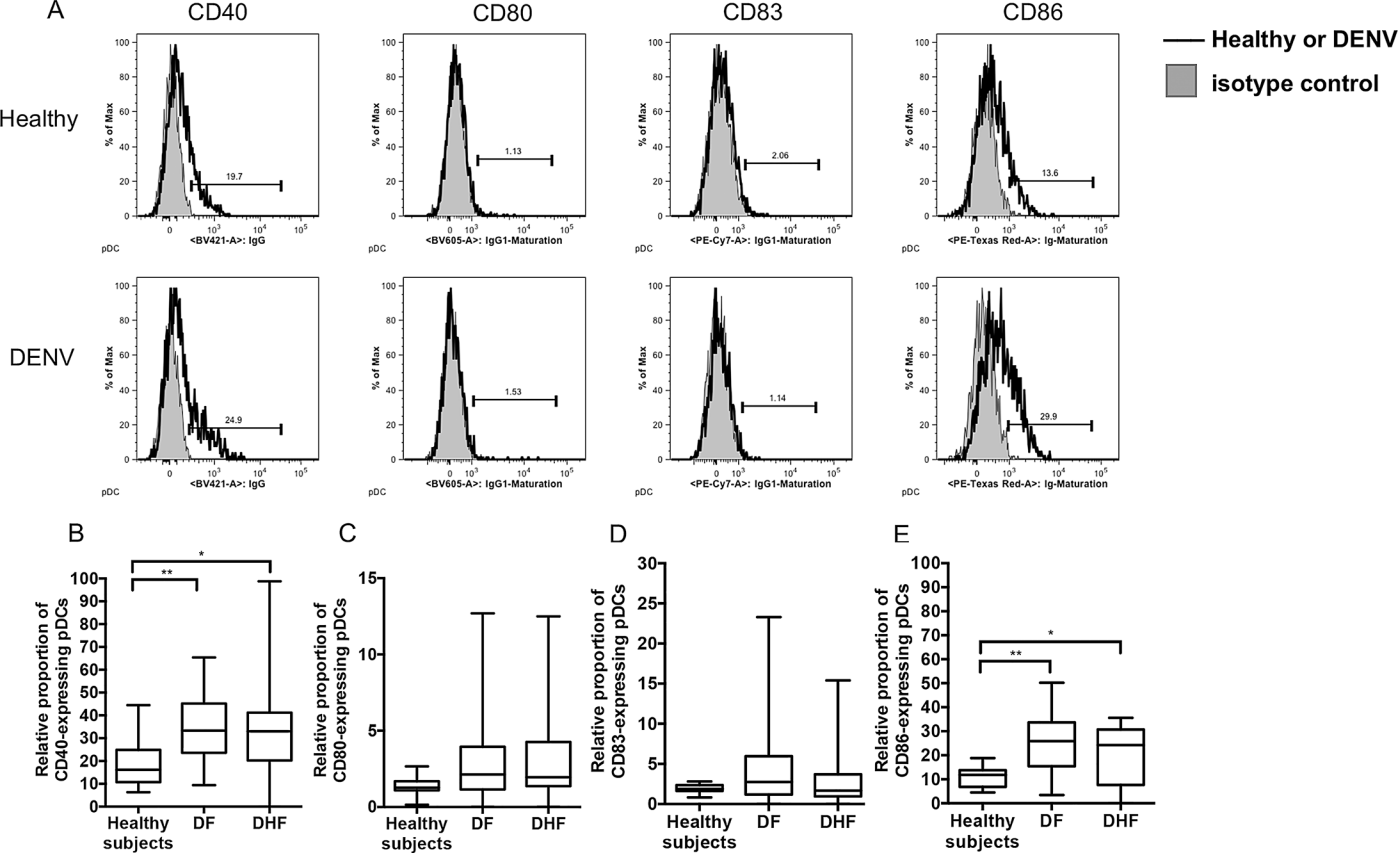
Comparison of the expression of potential markers of relative pDCs maturation during acute DENV infection. (A) Representative histogram plots showing expression of the potential maturation markers on pDCs in blood samples from healthy subjects (solid black line, upper panel) and DENV-infected patients (solid black line, lower panel). The shaded grey histograms represent profiles obtained using isotype controls. The relative proportion of CD40 (B), CD80 (C), CD83 (D), and CD86 (E) -expressing pDCs were compared between healthy subjects and DENV-infected patients of both DF and DHF patients. The box plot shows the median value (horizontal line in the box). The box and whisker represent 25^th^ to 75^th^, and 10^th^ to 90^th^ interquartile range, respectively. P values were determined by the Dunn’s post-test after Kruskal-Wallis test for comparison of three groups. (* p < 0.05 and ** p < 0.01).

We also studied the levels of maturation of pDCs during acute DENV infection. The representative histogram plots of the expression levels of the co-stimulatory molecules, CD40, CD80, CD83, and CD86 on pDCs are shown (Fig 6A). Although there was no difference in the relative proportion of pDC subset between healthy and DENV-infected patients, the relative proportion of CD40- and CD86- expressing pDCs in both DF and DHF patients were significantly higher than those of healthy individuals (Fig 6B and 6E). Interestingly, the MFI of CD40- and CD86 -expressing pDCs in DENV-infected patients were significantly higher than those of healthy individuals (S5B Fig). There was no significant difference in the relative proportion of CD80- and CD83 -expressing pDCs between healthy individuals and DENV-infected patients, DF and DHF patients (Fig 6C and 6D).

### Kinetic changes of DCs during acute DENV infection

**Fig 7:**
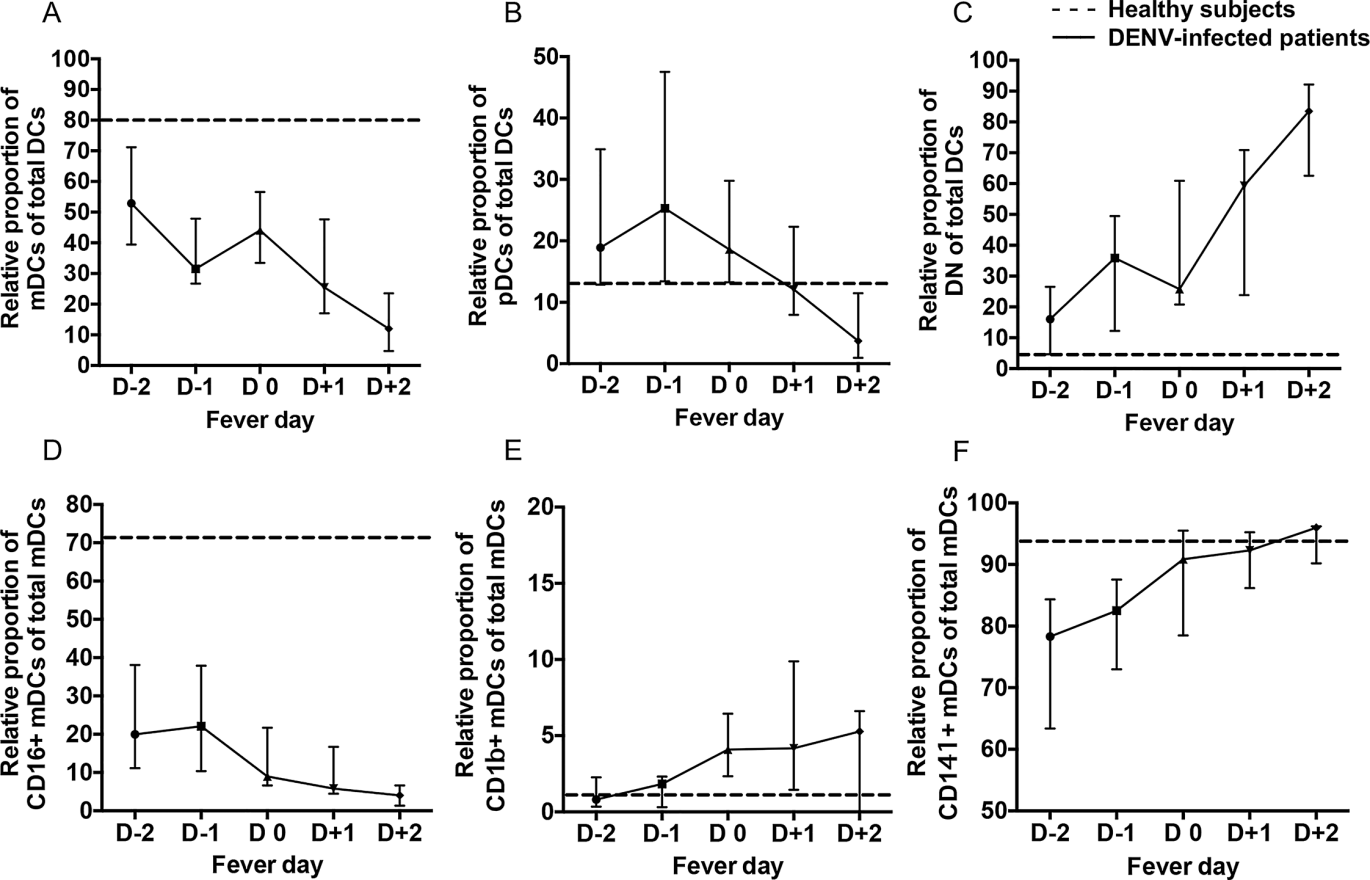
Kinetic changes of DC subsets during acute DENV infection. The kinetics of the relative proportion of mDCs (A), pDCs (B), DN (C), CD16^+^ mDCs (D), CD1b^+^ mDCs (E), and CD141^+^ mDCs (F) on samples from DENV-infected patients were determined at different days of fever ranging from febrile day-2 (D-2) and day-1 (D-1) to defervescence day 0 (D0) and day+1 (D+1) and to afebrile day+2 (D+2). Data are median and interquartile range. P values were determined by the Dunn’s post-test after Kruskal-Wallis test for comparison of the three groups. The median of relative proportion on samples from healthy subjects are denoted by a dashed line.

The altered relative proportion and phenotype of DCs during acute DENV infection prompted us to comparatively investigate in more details the kinetics of these changes among the DENV patients and healthy individuals. The kinetics of the relative proportion of DCs was compared based on day of defervescence (D0). The kinetic of DC subsets, mDCs, pDCs and DN were examined from D-2 to D+2 (Fig 7A–7C). As shown in Fig 7A, the relative proportion of total mDCs from DENV-infected patients were significantly (p<0.01) decreased throughout the course of infection from D-2 (febrile phase) to D+2 (afebrile phase). The relative proportion of total pDCs from DENV-infected patients were gradually increased in samples from the febrile phase and then significantly (p = 0.03) decreased from D-1 (febrile phase) to D+2 (afebrile phase) (Fig 7B). In contrast, the relative proportion of DN from DENV-infected patients were significantly (p<0.01) increased from the fever phase to the afebrile phase (Fig 7C).

We next extended this analysis by determining the kinetic changes in the relative proportion of mDC subsets during DENV infection. The kinetics of the relative proportion of CD16^+^, CD1b^+^ and CD141^+^ mDC subsets were compared in relation to the day of defervescence, described above. The relative proportion of CD16^+^ mDCs from DENV-infected patients were significantly (p = 0.04) decreased from D-1 (febrile phase) to D+2 (afebrile phase) (Fig 7D). In contrast, the relative proportion of CD1b^+^ mDCs were significantly (p = 0.02) increased from D-2 (febrile phase) to D0 (defervescence phase) (Fig 7E). The relative proportion of CD141^+^ mDCs from the DENV-infected patients were decreased during the early phase and then increased from the defervescence phase to the afebrile phase (Fig 7F).

**Fig 8:**
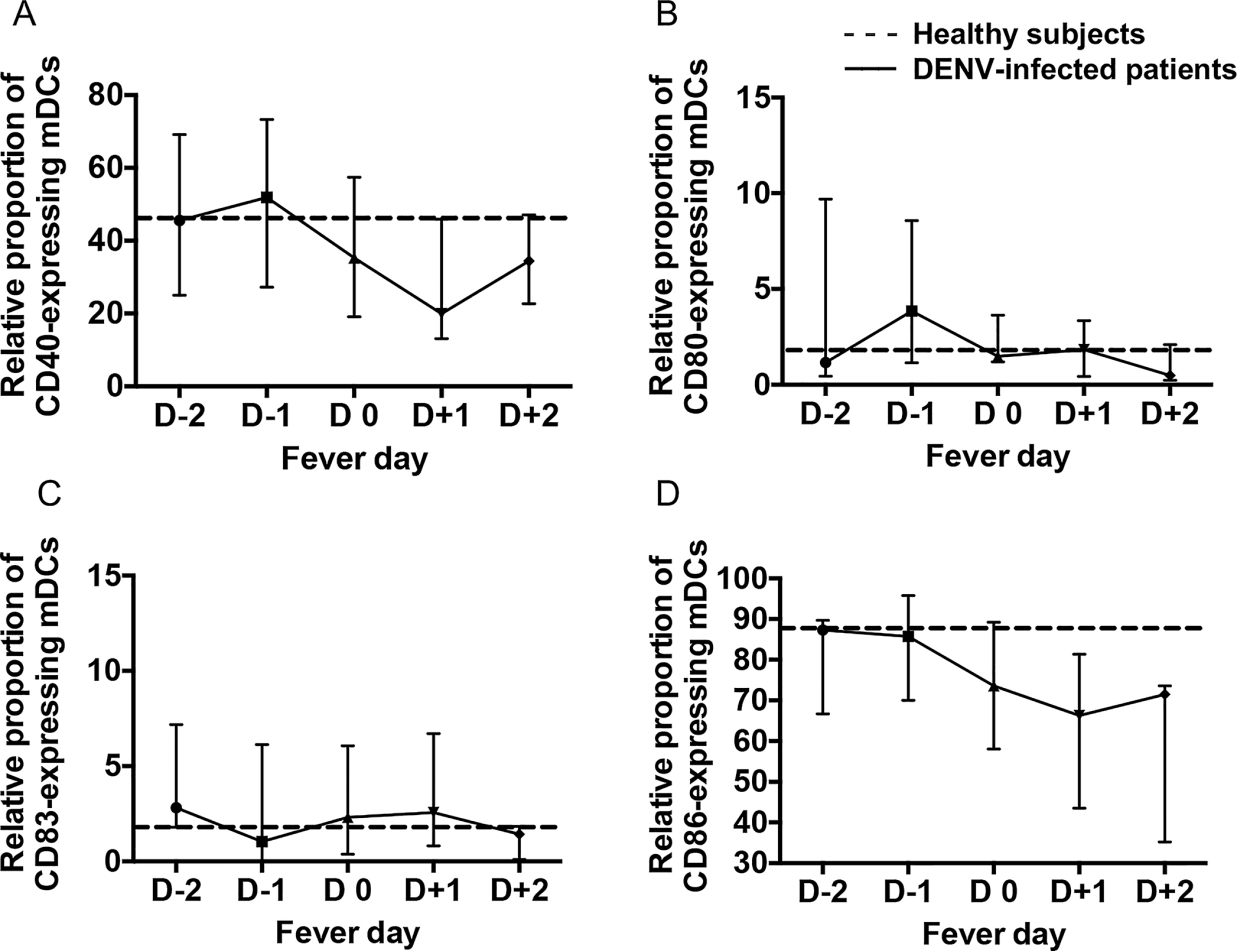
Kinetic changes of the maturation markers on mDCs during acute DENV infection. Expression of CD40 (A), CD80 (B), CD83 (C) and CD86 (D) -expressing mDCs were determined at different days of fever ranging from febrile day-2 (D-2) and day-1 (D-1) to defervescence day 0 (D0) and day+1 (D+1) and to afebrile day+2 (D+2). Data are median and interquartile range. P values were determined by the Dunn’s post-test after Kruskal-Wallis test for comparison of the three groups. The median of relative proportion on samples from healthy subjects are denoted by a dashed line.

Finally, we also investigated the kinetics of changes in the relative proportion of mDCs that expressed CD40, CD80, CD83 and CD86. Although there was no significant difference in the kinetics in the relative proportion of mDCs that expressed CD40, CD80, CD83 and CD86 in DENV-infected patients between days of fever (Fig 8A–8D), the relative proportion of CD40-expressing mDCs from DENV-infected patients gradually increased from the febrile phase and then decreased to below normal level from D-1 to D+1 and returned to normal levels during the afebrile phase (Fig 8A). The relative proportion of CD86-expressing mDCs from DENV-infected patients, on the other hand decreased from the febrile phase to the afebrile phase when compared with values on samples from healthy individuals (Fig 8D). Moreover, there was no significant difference in the kinetics in the MFI of mDCs that expressed CD40, CD80, CD83 and CD86 in DENV-infected patients between days of fever (S6 Fig). These data suggest that there are changes in the subsets of DCs that are secondary to stages of the disease in dengue viral infection.

## Discussion

DCs play a key role in priming innate and adaptive immune responses against microbial infection such as viral infections. In the case of responses to DENV infection, host cells recognize DENV associated PAMPs and activate TLRs, leading to the initiation of antiviral immune responses that include the synthesis of IFN-α/β and a number of pro-inflammatory cytokines [25, 26]. Several studies on various aspects of DCs during DENV infection have been reported both in adult [21] and children [22] but as noted above, most of these studies were cross sectional and did not address which of the subsets of DCs contributed to the observations that were reported. In an effort to better understand the role of DCs, we characterized the relative proportion, phenotypic changes and maturation status of both total mDCs/pDCs and subsets of mDCs in the peripheral blood from children who had DENV infection.

Our study showed that while dengue infection did not change the relative proportion of pDCs, the infection led to a significant decrease in the relative proportion of mDCs. Importantly, since the relative proportion of mDCs in children with DHF were significantly lower than DF patients, the data suggest that decreased levels of mDCs correlate with an increase in disease severity. These data were consistent with the previous work in children with DENV infection [22]. The possible mechanisms that could contribute to a decrease in the number of circulating mDCs include a) increased migration of mDCs to peripheral tissues or lymphoid organs b) impairment of bone marrow function and c) increased apoptosis of mDCs. Support for differences in trafficking of DC’s comes from studies that have documented the fact that mDCs residing within epithelial tissues synthesize significant levels of TNF-α, IFN- α and IL-6 [12, 27] along with inflammatory chemokines upon encounter with dengue viral antigens which promotes their migration from the blood to sites of inflammation [28]. Such trafficking of circulating DCs to secondary lymphoid organs has also been noted in both human HIV [29] and nonhuman primate SIV infection [30]. As previously reported, DENV has been shown to infect cells within the bone marrow. It is therefore clearly possible that such infection leads to reduced progenitor cell development resulting in reduced levels of DCs [31]. Finally, support for a potential role for increased levels of apoptosis comes from the studies that have documented increase levels of apoptotic cells in the liver, brain, intestinal/lung tissues, and microvascular endothelial cells from fatal cases of DHF/DSS [13]. It has been shown that during DENV infection, there is an accumulation of DENV proteins within the endoplasmic reticulum (ER). Thus, such accumulation may be one of the mechanisms that induces ER stress leading to the activation of the apoptotic pathway [32, 33]. Interestingly, there was no significant difference in the relative proportion of pDCs between DENV-infected patients and healthy controls except for the kinetic change noted in the relative proportion of pDCs that were increased during the early febrile phase when compared with that of healthy individuals. We submit that the ability to maintain significant levels of pDCs levels may contribute to viral clearance during acute DENV infection.

In addition, we observed an increase in the relative proportion of Lin^-^HLA-DR^+^CD11c^-^CD123^-^ cells (DN subset) in children with DHF but not DF. This DN subset has been previously identified as Lin^-^HLA-DR^+^CD34^+^ cells [14, 34]. The expression of CD34 has been shown to be a marker of circulating progenitor cells [35]. Thus, DCs develop from CD34^+^ hematopoietic progenitor cells (HPCs) which are mostly found in the bone marrow and are present in very small numbers in the peripheral blood [36]. It is therefore tempting to speculate that the increased levels of this DN subset in dengue infection could be a reflection of a hematopoietic response to decreased levels of mDCs that induces the maturation of precursor cells. Unfortunately, we did not include analysis of CD34 expression in our study, but is a subject of current studies.

Human mDC subsets are subdivided into 3 subsets based on the expression of CD16^+^, CD1c^+^ (BDCA-1^+^) or CD141^+^ (BDCA-3^+^) [14, 16, 17]. In the present study, we characterized these three mDC subsets by their expression of these cell surface markers. The CD1b is a member of group 1 of the CD1 family of molecules similar to CD1c [37]. Both CD1b^+^ and CD1c^+^ have been shown to present microbial lipid antigen to T cells [38–40] and are classified as non-classical antigen presenting molecules and that CD1b^+^ expressing DCs play a major role in the synthesis of select cytokines such as IL-10 and IFN-γ and promote the development of Th1 cells [41]. We demonstrated herein that CD16^+^ and CD141^+^ and mDC subsets were the major mDC subsets in healthy individuals. Of interest was our findings of significant decreases in the relative proportion of both CD16^+^ and CD141^+^ mDC subsets during acute DENV infection when compared with those of healthy individuals. The kinetic changes of relative proportion of CD16^+^ and CD141^+^ mDCs were different. While CD16^+^ mDCs were continuously decreased from febrile to the afebrile phase, CD141^+^ mDCs were decreased during the early febrile phase. Previous studies have shown that the CD16^+^ mDCs play a key role in innate immunity by secretion of chemoattractant stimuli and cytokines as well as playing a role in the induction of inflammation [16]. The CD141^+^ mDCs on the other hand have been shown to play a crucial role in induction of CD8^+^ T cells by cross-presentation of antigen to CD8^+^ T cells [19, 42]. Our data suggest that decreased relative proportion of CD16^+^ and CD141^+^ mDC subsets in DENV patients may limit their capacities in executing both innate and adaptive immune responses.

CD33 (Siglec-3) consists of two Ig-like extracellular domains and two immune-receptor tyrosine-based inhibitory (ITIM)-like motifs within the cytoplasmic domain [43, 44]. CD33 is predominantly expressed by cells of the myeloid lineage and appears during the early stages of myeloid cell differentiation and on normal myeloid pre-progenitor cells. The data presented herein demonstrated that there was a significant decrease in the level of CD33 expressed by mDCs especially CD16^+^ mDCs in DENV patients suggesting a possible impairment in the generation of DCs and myeloid cell maturation. Results of a previous study demonstrated that DENV induces an increase in the overall maturation of mDCs as defined by increased levels of expression of the co-stimulatory molecules CD40, CD80, CD83, and CD86 [12]. It is important to note, however, that infected DCs but not innocent bystander cells are inhibited from undergoing maturation in the presence of DENV infection [45]. The decreased relative proportion but increased expression level (MFI) of CD33-expressing mDCs in DENV patients prompted us to determine whether such decreases led to an impairment in mDC maturation. Interestingly, while there were not globally changed in the MFI of the maturation profiles of mDCs, there was a decrease in the relative proportion of CD86-expressing mDCs. These data suggest that the relative proportion of CD33-expressing mDCs may not be related to the maturation status of mDCs during acute DENV infection and that functional effect of CD33 on maturation of DC is insufficient for predicting maturation status. Future studies involving functional analysis of CD33 on mDCs in DENV patients may be necessary. Of note, we demonstrated that the relative proportion of pDCs did not change significantly, but DENV induces the maturation of pDCs by the up-regulation of CD40 and CD86 markers in children with DF and DHF. On the other hand, pDCs showed down-regulation of CD86, a co-stimulatory marker in adults with DF and DHF [25]. These findings suggest there may be differences in the maturation profile of DCs between children and adult.

In conclusion, our study provides a detailed characterization of mDCs and pDCs, and their maturation levels during DENV disease. We have shown that a reduction in the relative proportion of mDCs is associated with an increase in disease severity. The decrease is markedly shown in the two major mDC subsets which include the CD16^+^ and CD141^+^ mDCs subsets. DENV infection also upregulates the maturation of pDCs and alters the maturation status of mDCs. These findings have implication for better understanding the phenotypic changes of DCs and their maturation kinetics during the febrile phase of DENV disease.

## Supporting information

S1 Fig Gating strategy for the analysis of DCs from peripheral blood of healthy individual. Two major populations of DC subsets were identified from CD45^+^ cells followed by CD16 of both large and small cells and those with different levels of granularity. The CD45^+^/CD14^-^/CD3^-^/CD19^-^/CD7^-^/HLA-DR^+^ cells were further analysed for mDCs as identified by their CD11c^+^CD123^lo^ and pDCs which were identified as CD11c^-^CD123^+^. The percentage of double negative (DN) subset (CD11c^-^CD123^-^) were also noted.
(TIF)

S2 Fig Gating strategy for mDC subsets in peripheral blood. Dot plots show three populations of mDC subsets. These include the CD16^+^, CD1b^+^ and CD141^+^ mDCs on the gated population of CD45^+^/CD14^-^/CD3^-^/CD19^-^/CD56^-^/HLA-DR^+^/CD304^-^/CD11C^+^/CD123^-^.
(TIF)

S3 Fig Gating strategy for CD33 expressing-mDCs and -pDCs. Representative histogram plots showing maturation markers on mDCs (A) and pDCs (B) on blood samples from DENV-infected patient and healthy individual (solid black line) and isotype control (shaded grey).
(TIF)

S4 Fig Comparison of the CD33-expressing CD1b^+^ mDCs and CD141^+^ mDCs in DENV infected patients. The relative proportion of CD33-expressing CD1b^+^ (A) and CD141^+^ (B) mDCs in DENV-infected patients were compared with healthy individuals. The box plot shows the median value (horizontal line in the box). The box and whisker represent 25^th^ to 75^th^, and 10^th^ to 90^th^ interquartile range, respectively. P values were determined by the Mann-Whitney U test for comparison between two groups.
(TIF)

S5 Fig Comparison of the MFI of maturation profiles on mDCs and pDCs in DENV infected patients. The MFI of CD40, CD80, CD83, and CD86 -expressing mDCs (A) and pDCs (B) were compared on samples from healthy subjects and DENV-infected patients of both DF and DHF patients. The box plot shows the median value (horizontal line in the box). The box and whisker represent 25^th^ to 75^th^, and 10^th^ to 90^th^ interquartile range, respectively. P values were determined by the Dunn’s post-test after Kruskal-Wallis test for comparison of three groups. (* p < 0.05 and ** p < 0.01).
(TIF)

S6 Fig Kinetic changes of the MFI of maturation markers on mDCs during acute DENV infection. The MFI of CD40 (A), CD80 (B), CD83 (C) and CD86 (D) -expressing mDCs were determined at different days of fever ranging from febrile day-2 (D-2) and day-1 (D-1) to defervescence day 0 (D0) and day+1 (D+1) and to afebrile day+2 (D+2). Data are median and interquartile range. P values were determined by the Dunn’s post-test after Kruskal-Wallis test for comparison of three groups. The median of MFI on samples from healthy subjects are denoted by a dashed line.
(TIF)

S1 Table Primer sequences for amplification of dengue viral RNA. (TIF)

S2 Table Surface staining panels for the phenotypic characterization of DC. (TIF)

## References

[1] Chambers TJ, Tsai TF, Pervikov Y, Monath TP. Vaccine development against dengue and Japanese encephalitis: report of a World Health Organization meeting. Vaccine. 1997;15(14):1494–502. . 9330458 10.1016/s0264-410x(97)00195-3

[2] Whitehead SS, Blaney JE, Durbin AP, Murphy BR. Prospects for a dengue virus vaccine. Nat Rev Microbiol. 2007;5(7):518–28. 10.1038/nrmicro1690 . 17558424

[3] Bhatt S, Gething PW, Brady OJ, Messina JP, Farlow AW, Moyes CL, et al The global distribution and burden of dengue. Nature. 2013;496(7446):504–7. 10.1038/nature12060 ; PubMed Central PMCID: PMCPMC3651993. 23563266 PMC3651993

[4] Halstead SB, Cohen SN. Dengue Hemorrhagic Fever at 60 Years: Early Evolution of Concepts of Causation and Treatment. Microbiol Mol Biol Rev. 2015;79(3):281–91. 10.1128/MMBR.00009-15 ; PubMed Central PMCID: PMCPMC4488372. 26085471 PMC4488372

[5] Hermann LL, Gupta SB, Manoff SB, Kalayanarooj S, Gibbons RV, Coller BA. Advances in the understanding, management, and prevention of dengue. J Clin Virol. 2015;64:153–9. 10.1016/j.jcv.2014.08.031 . 25453329

[6] Halstead SB, O'Rourke EJ. Antibody-enhanced dengue virus infection in primate leukocytes. Nature. 1977;265(5596):739–41. . 404559 10.1038/265739a0

[7] Goncalvez AP, Engle RE, St Claire M, Purcell RH, Lai CJ. Monoclonal antibody-mediated enhancement of dengue virus infection in vitro and in vivo and strategies for prevention. Proc Natl Acad Sci U S A. 2007;104(22):9422–7. 10.1073/pnas.0703498104 ; PubMed Central PMCID: PMCPMC1868655. 17517625 PMC1868655

[8] Guzman MG, Alvarez M, Halstead SB. Secondary infection as a risk factor for dengue hemorrhagic fever/dengue shock syndrome: an historical perspective and role of antibody-dependent enhancement of infection. Arch Virol. 2013;158(7):1445–59. 10.1007/s00705-013-1645-3 . 23471635

[9] Bonasio R, von Andrian UH. Generation, migration and function of circulating dendritic cells. Curr Opin Immunol. 2006;18(4):503–11. 10.1016/j.coi.2006.05.011 . 16777395

[10] Wu SJ, Grouard-Vogel G, Sun W, Mascola JR, Brachtel E, Putvatana R, et al Human skin Langerhans cells are targets of dengue virus infection. Nat Med. 2000;6(7):816–20. 10.1038/77553 . 10888933

[11] Ho LJ, Wang JJ, Shaio MF, Kao CL, Chang DM, Han SW, et al Infection of human dendritic cells by dengue virus causes cell maturation and cytokine production. J Immunol. 2001;166(3):1499–506. . 11160189 10.4049/jimmunol.166.3.1499

[12] Libraty DH, Pichyangkul S, Ajariyakhajorn C, Endy TP, Ennis FA. Human dendritic cells are activated by dengue virus infection: enhancement by gamma interferon and implications for disease pathogenesis. J Virol. 2001;75(8):3501–8. 10.1128/JVI.75.8.3501-3508.2001 ; PubMed Central PMCID: PMCPMC114841. 11264339 PMC114841

[13] Limonta D, Capo V, Torres G, Perez AB, Guzman MG. Apoptosis in tissues from fatal dengue shock syndrome. J Clin Virol. 2007;40(1):50–4. 10.1016/j.jcv.2007.04.024 . 17693133

[14] MacDonald KP, Munster DJ, Clark GJ, Dzionek A, Schmitz J, Hart DN. Characterization of human blood dendritic cell subsets. Blood. 2002;100(13):4512–20. 10.1182/blood-2001-11-0097 . 12393628

[15] Robinson SP, Patterson S, English N, Davies D, Knight SC, Reid CD. Human peripheral blood contains two distinct lineages of dendritic cells. Eur J Immunol. 1999;29(9):2769–78. 10.1002/(SICI)1521-4141(199909)29:09<2769::AID-IMMU2769>3.0.CO;2-2 . 10508251

[16] Piccioli D, Tavarini S, Borgogni E, Steri V, Nuti S, Sammicheli C, et al Functional specialization of human circulating CD16 and CD1c myeloid dendritic-cell subsets. Blood. 2007;109(12):5371–9. 10.1182/blood-2006-08-038422 . 17332250

[17] Dzionek A, Fuchs A, Schmidt P, Cremer S, Zysk M, Miltenyi S, et al BDCA-2, BDCA-3, and BDCA-4: three markers for distinct subsets of dendritic cells in human peripheral blood. J Immunol. 2000;165(11):6037–46. . 11086035 10.4049/jimmunol.165.11.6037

[18] Porcelli SA, Segelke BW, Sugita M, Wilson IA, Brenner MB. The CD1 family of lipid antigen-presenting molecules. Immunol Today. 1998;19(8):362–8. . 9709504 10.1016/s0167-5699(98)01289-4

[19] Jongbloed SL, Kassianos AJ, McDonald KJ, Clark GJ, Ju X, Angel CE, et al Human CD141+ (BDCA-3)+ dendritic cells (DCs) represent a unique myeloid DC subset that cross-presents necrotic cell antigens. J Exp Med. 2010;207(6):1247–60. 10.1084/jem.20092140 ; PubMed Central PMCID: PMCPMC2882828. 20479116 PMC2882828

[20] Gandini M, Gras C, Azeredo EL, Pinto LM, Smith N, Despres P, et al Dengue virus activates membrane TRAIL relocalization and IFN-alpha production by human plasmacytoid dendritic cells in vitro and in vivo. PLoS Negl Trop Dis. 2013;7(6):e2257 10.1371/journal.pntd.0002257 ; PubMed Central PMCID: PMCPMC3675005. 23755314 PMC3675005

[21] De Carvalho Bittencourt M, Martial J, Cabie A, Thomas L, Cesaire R. Decreased peripheral dendritic cell numbers in dengue virus infection. J Clin Immunol. 2012;32(1):161–72. 10.1007/s10875-011-9592-9 . 21969208

[22] Pichyangkul S, Endy TP, Kalayanarooj S, Nisalak A, Yongvanitchit K, Green S, et al A blunted blood plasmacytoid dendritic cell response to an acute systemic viral infection is associated with increased disease severity. J Immunol. 2003;171(10):5571–8. . 14607965 10.4049/jimmunol.171.10.5571

[23] Perdomo-Celis F, Romero F, Salgado DM, Vega R, Rodriguez J, Angel J, et al Identification and characterization at the single-cell level of cytokine-producing circulating cells in children with dengue. J Infect Dis. 2018 Epub 2018/02/02. 10.1093/infdis/jiy053 . 29390091

[24] Crespo HJ, Cabral MG, Teixeira AV, Lau JT, Trindade H, Videira PA. Effect of sialic acid loss on dendritic cell maturation. Immunology. 2009;128(1 Suppl):e621–31. Epub 2009/09/25. 10.1111/j.1365-2567.2009.03047.x ; PubMed Central PMCID: PMCPMC2753891. 19740323 PMC2753891

[25] Torres S, Hernandez JC, Giraldo D, Arboleda M, Rojas M, Smit JM, et al Differential expression of Toll-like receptors in dendritic cells of patients with dengue during early and late acute phases of the disease. PLoS Negl Trop Dis. 2013;7(2):e2060 10.1371/journal.pntd.0002060 ; PubMed Central PMCID: PMCPMC3585035. 23469297 PMC3585035

[26] Tsai YT, Chang SY, Lee CN, Kao CL. Human TLR3 recognizes dengue virus and modulates viral replication in vitro. Cell Microbiol. 2009;11(4):604–15. Epub 2009/01/13. 10.1111/j.1462-5822.2008.01277.x . 19134117

[27] Sun P, Fernandez S, Marovich MA, Palmer DR, Celluzzi CM, Boonnak K, et al Functional characterization of ex vivo blood myeloid and plasmacytoid dendritic cells after infection with dengue virus. Virology. 2009;383(2):207–15. 10.1016/j.virol.2008.10.022 . 19013627

[28] Penna G, Sozzani S, Adorini L. Cutting edge: selective usage of chemokine receptors by plasmacytoid dendritic cells. J Immunol. 2001;167(4):1862–6. . 11489962 10.4049/jimmunol.167.4.1862

[29] Hosmalin A, Lichtner M, Louis S. Clinical analysis of dendritic cell subsets: the dendritogram. Methods Mol Biol. 2008;415:273–90. 10.1007/978-1-59745-570-1_16 . 18370160

[30] Diop OM, Ploquin MJ, Mortara L, Faye A, Jacquelin B, Kunkel D, et al Plasmacytoid dendritic cell dynamics and alpha interferon production during Simian immunodeficiency virus infection with a nonpathogenic outcome. J Virol. 2008;82(11):5145–52. 10.1128/JVI.02433-07 ; PubMed Central PMCID: PMCPMC2395206. 18385227 PMC2395206

[31] La Russa VF, Innis BL. Mechanisms of dengue virus-induced bone marrow suppression. Baillieres Clin Haematol. 1995;8(1):249–70. . 7663049 10.1016/s0950-3536(05)80240-9

[32] Marianneau P, Cardona A, Edelman L, Deubel V, Despres P. Dengue virus replication in human hepatoma cells activates NF-kappaB which in turn induces apoptotic cell death. J Virol. 1997;71(4):3244–9. ; PubMed Central PMCID: PMCPMC191457. 9060688 10.1128/jvi.71.4.3244-3249.1997 PMC191457

[33] Despres P, Flamand M, Ceccaldi PE, Deubel V. Human isolates of dengue type 1 virus induce apoptosis in mouse neuroblastoma cells. J Virol. 1996;70(6):4090–6. ; PubMed Central PMCID: PMCPMC190291. 8648748 10.1128/jvi.70.6.4090-4096.1996 PMC190291

[34] Fromm PD, Kupresanin F, Brooks AE, Dunbar PR, Haniffa M, Hart DN, et al A multi-laboratory comparison of blood dendritic cell populations. Clin Transl Immunology. 2016;5(4):e68 10.1038/cti.2016.5 ; PubMed Central PMCID: PMCPMC4855271. 27195111 PMC4855271

[35] Kondo M, Wagers AJ, Manz MG, Prohaska SS, Scherer DC, Beilhack GF, et al Biology of hematopoietic stem cells and progenitors: implications for clinical application. Annu Rev Immunol. 2003;21:759–806. 10.1146/annurev.immunol.21.120601.141007 . 12615892

[36] Wu L, Liu YJ. Development of dendritic-cell lineages. Immunity. 2007;26(6):741–50. 10.1016/j.immuni.2007.06.006 . 17582346

[37] Siddiqui S, Visvabharathy L, Wang CR. Role of Group 1 CD1-Restricted T Cells in Infectious Disease. Front Immunol. 2015;6:337 10.3389/fimmu.2015.00337 ; PubMed Central PMCID: PMCPMC4484338. 26175733 PMC4484338

[38] Cala-De Paepe D, Layre E, Giacometti G, Garcia-Alles LF, Mori L, Hanau D, et al Deciphering the role of CD1e protein in mycobacterial phosphatidyl-myo-inositol mannosides (PIM) processing for presentation by CD1b to T lymphocytes. J Biol Chem. 2012;287(37):31494–502. 10.1074/jbc.M112.386300 ; PubMed Central PMCID: PMCPMC3438982. 22782895 PMC3438982

[39] Gilleron M, Stenger S, Mazorra Z, Wittke F, Mariotti S, Bohmer G, et al Diacylated sulfoglycolipids are novel mycobacterial antigens stimulating CD1-restricted T cells during infection with Mycobacterium tuberculosis. J Exp Med. 2004;199(5):649–59. 10.1084/jem.20031097 ; PubMed Central PMCID: PMCPMC2213295. 14981115 PMC2213295

[40] Beckman EM, Porcelli SA, Morita CT, Behar SM, Furlong ST, Brenner MB. Recognition of a lipid antigen by CD1-restricted alpha beta+ T cells. Nature. 1994;372(6507):691–4. 10.1038/372691a0 . 7527500

[41] Olivier M, Foret B, Le Vern Y, Kerboeuf D, Guilloteau LA. Plasticity of migrating CD1b+ and CD1b- lymph dendritic cells in the promotion of Th1, Th2 and Th17 in response to Salmonella and helminth secretions. PLoS One. 2013;8(11):e79537 Epub 2013/11/14. 10.1371/journal.pone.0079537 ; PubMed Central PMCID: PMCPMC3818231. 24223964 PMC3818231

[42] Bachem A, Guttler S, Hartung E, Ebstein F, Schaefer M, Tannert A, et al Superior antigen cross-presentation and XCR1 expression define human CD11c+CD141+ cells as homologues of mouse CD8+ dendritic cells. J Exp Med. 2010;207(6):1273–81. 10.1084/jem.20100348 ; PubMed Central PMCID: PMCPMC2882837. 20479115 PMC2882837

[43] Crocker PR, Varki A. Siglecs, sialic acids and innate immunity. Trends Immunol. 2001;22(6):337–42. . 11377294 10.1016/s1471-4906(01)01930-5

[44] Ulyanova T, Blasioli J, Woodford-Thomas TA, Thomas ML. The sialoadhesin CD33 is a myeloid-specific inhibitory receptor. Eur J Immunol. 1999;29(11):3440–9. 10.1002/(SICI)1521-4141(199911)29:11<3440::AID-IMMU3440>3.0.CO;2-C . 10556798

[45] Sun P, Celluzzi CM, Marovich M, Subramanian H, Eller M, Widjaja S, et al CD40 ligand enhances dengue viral infection of dendritic cells: a possible mechanism for T cell-mediated immunopathology. J Immunol. 2006;177(9):6497–503. . 17056582 10.4049/jimmunol.177.9.6497

