# Supplementary figures and images for: Identification of changes in dendritic cell subsets that correlate with disease severity in dengue infection
Source: PLoS One. 2018 Jul 12;13(7):e0200564. doi: 10.1371/journal.pone.0200564 (PMC6042784; doi:10.1371/journal.pone.0200564)

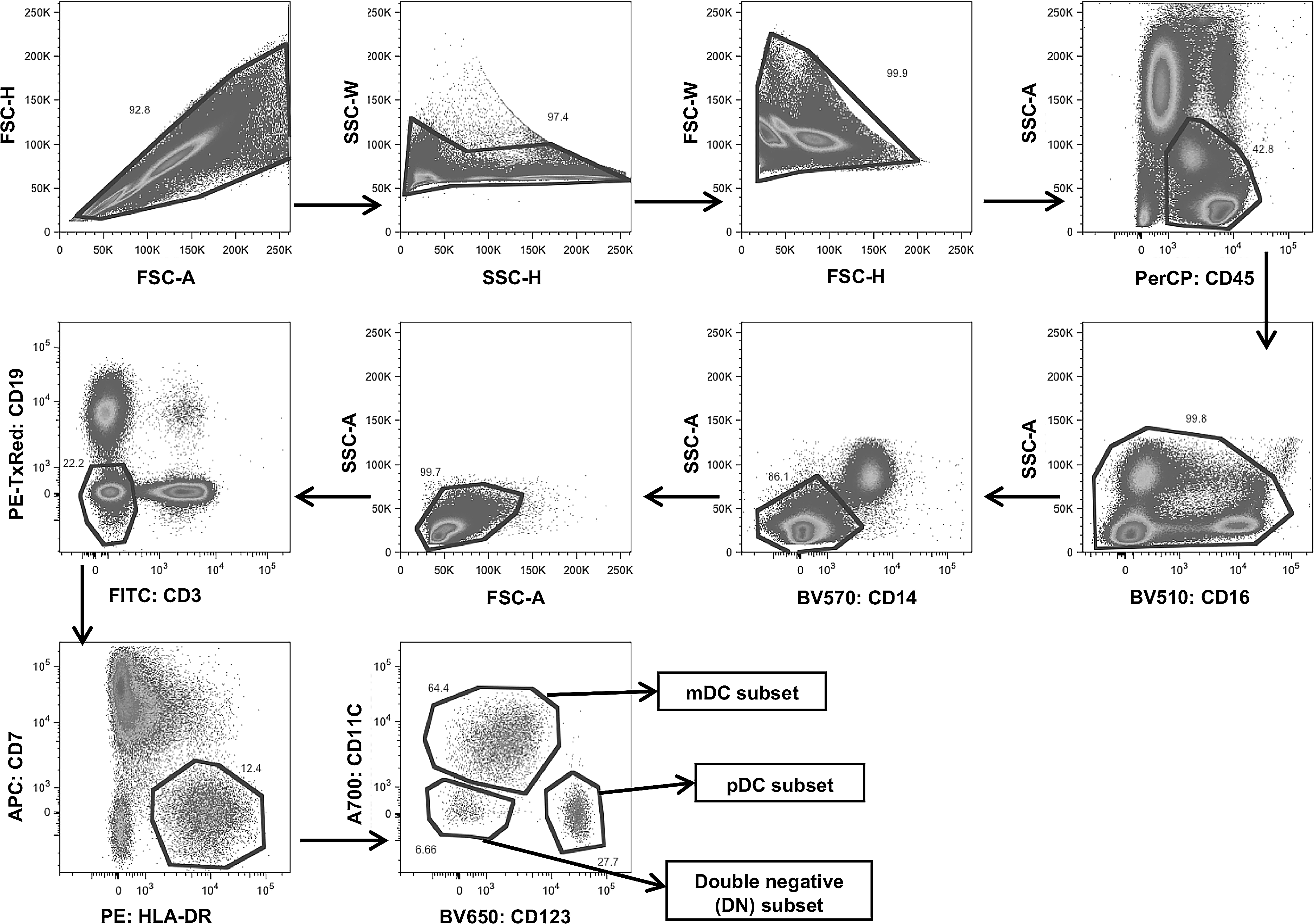

Supplement: S1 Fig — Two major populations of DC subsets were identified from CD45+ cells followed by CD16 of both large and small cells and those with different levels of granularity. The CD45+/CD14-/CD3-/CD19-/CD7-/HLA-DR+ cells were further analysed for mDCs as identified by their CD11c+CD123lo and pDCs which were identified as CD11c-CD123+. The percentage of double negative (DN) subset (CD11c-CD123-) were also noted. (TIF) [file pone.0200564.s001.tif]

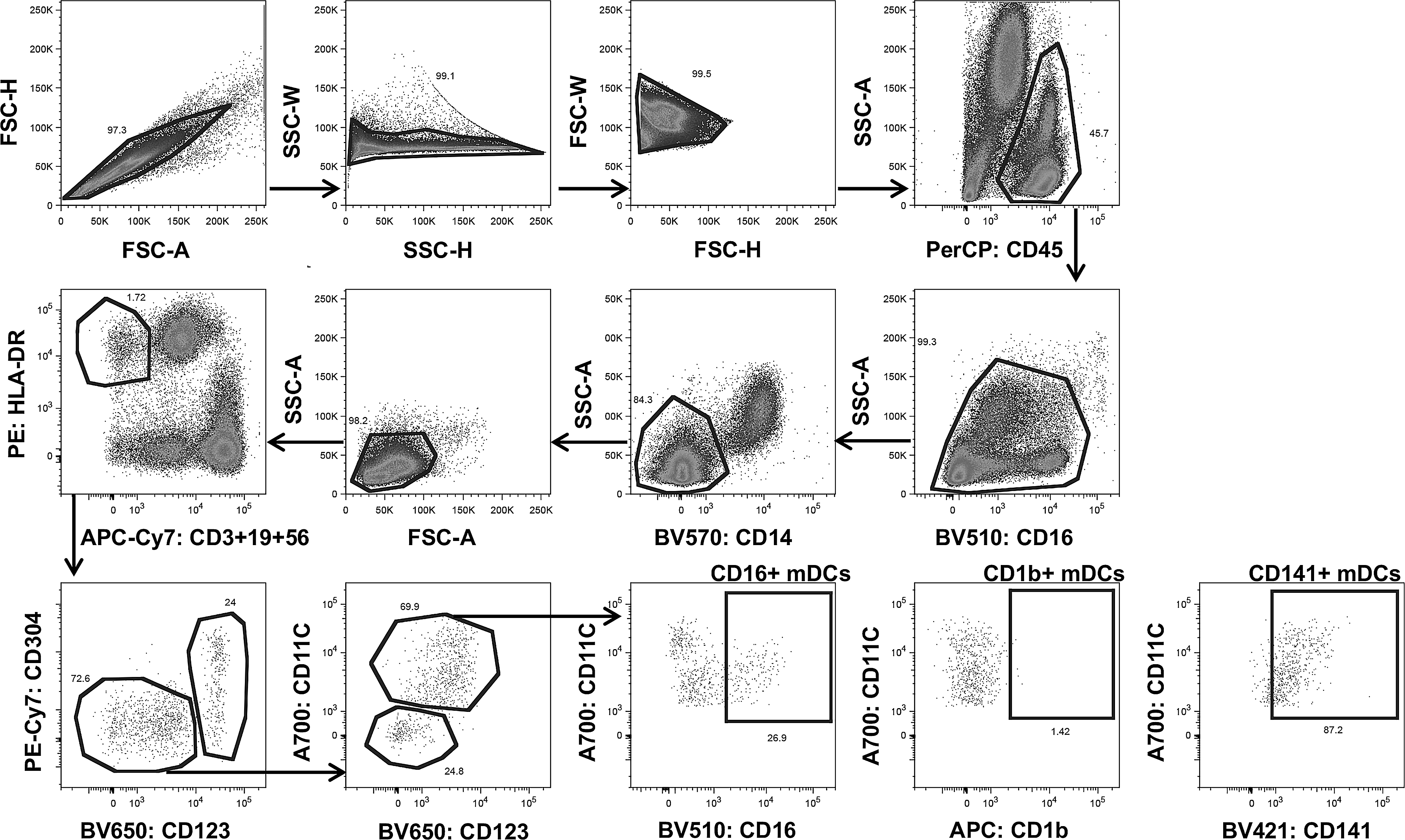

Supplement: S2 Fig — Dot plots show three populations of mDC subsets. These include the CD16+, CD1b+ and CD141+ mDCs on the gated population of CD45+/CD14-/CD3-/CD19-/CD56-/HLA-DR+/CD304-/CD11C+/CD123-. (TIF) [file pone.0200564.s002.tif]

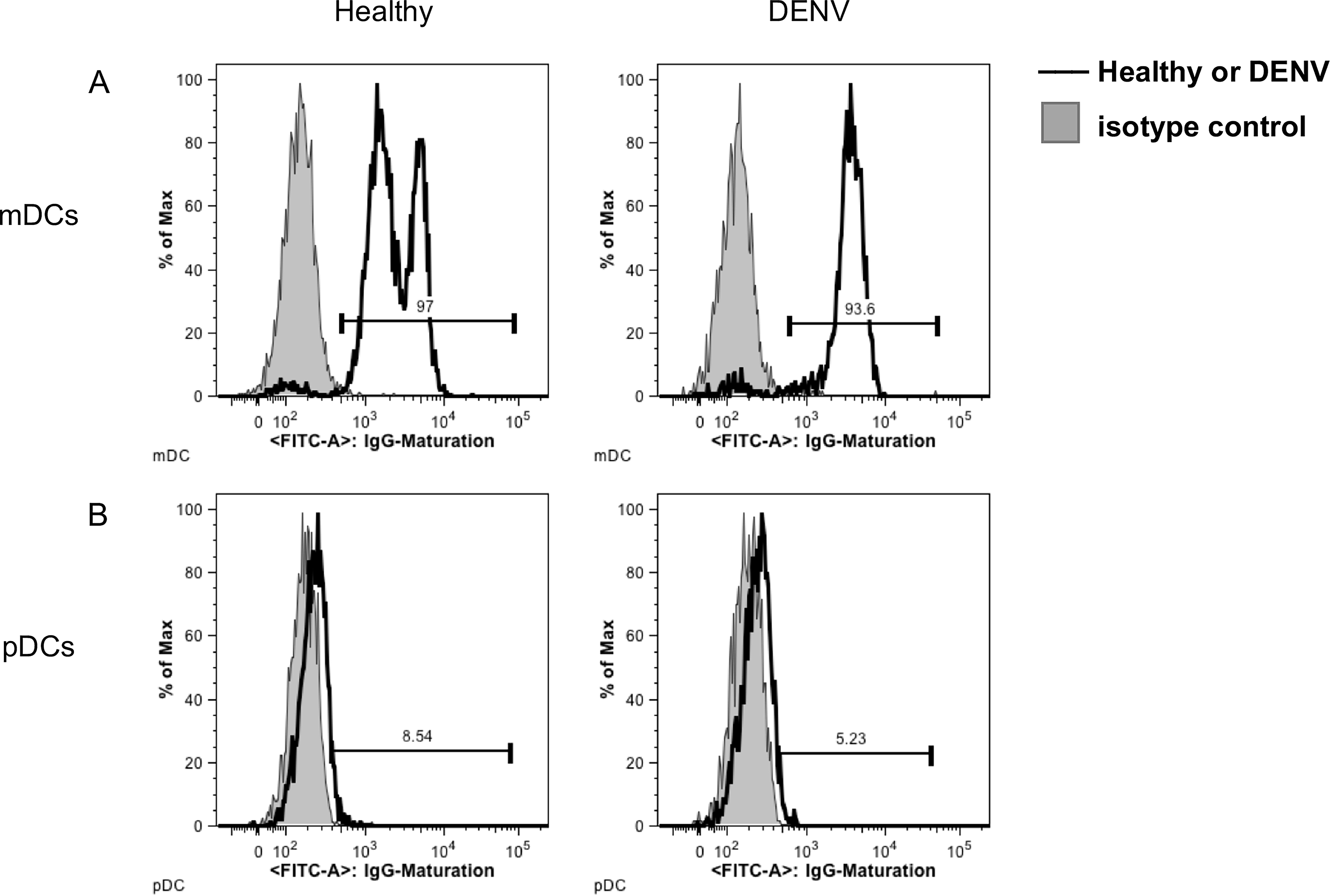

Supplement: S3 Fig — Representative histogram plots showing maturation markers on mDCs (A) and pDCs (B) on blood samples from DENV-infected patient and healthy individual (solid black line) and isotype control (shaded grey). (TIF) [file pone.0200564.s003.tif]

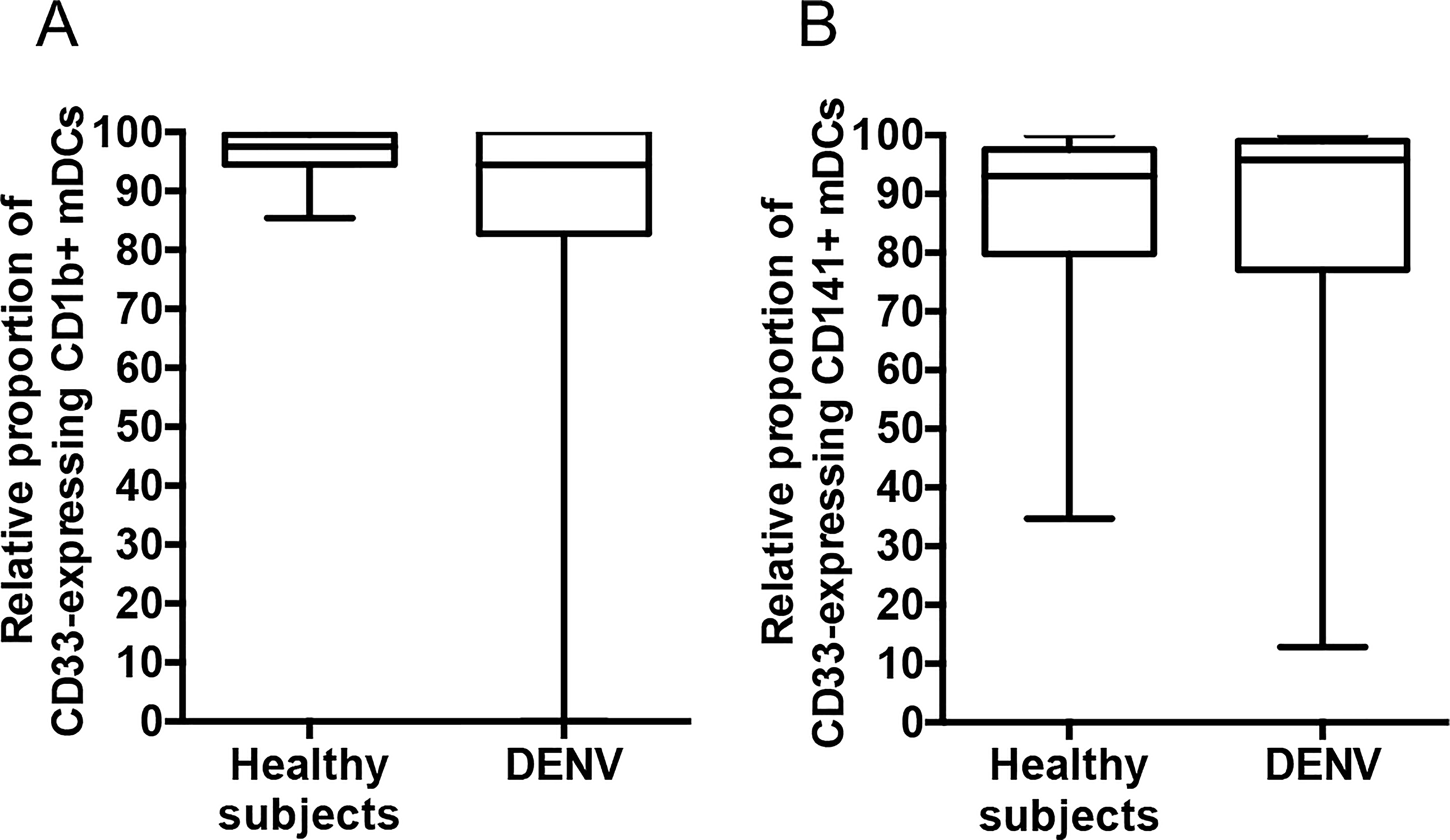

Supplement: S4 Fig — The relative proportion of CD33-expressing CD1b+ (A) and CD141+ (B) mDCs in DENV-infected patients were compared with healthy individuals. The box plot shows the median value (horizontal line in the box). The box and whisker represent 25th to 75th, and 10th to 90th interquartile range, respectively. P values were determined by the Mann-Whitney U test for comparison between two groups. (TIF) [file pone.0200564.s004.tif]

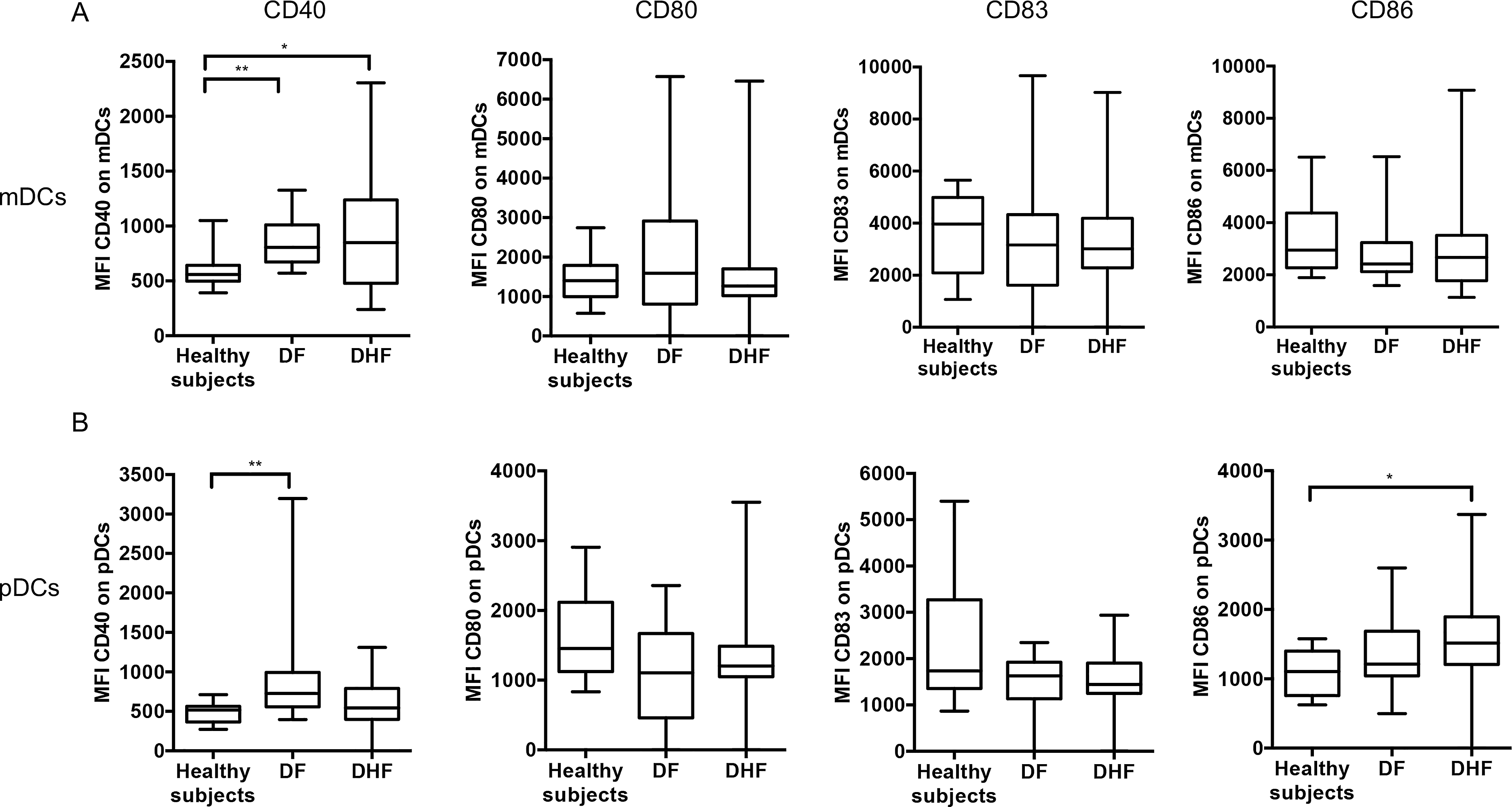

Supplement: S5 Fig — The MFI of CD40, CD80, CD83, and CD86 -expressing mDCs (A) and pDCs (B) were compared on samples from healthy subjects and DENV-infected patients of both DF and DHF patients. The box plot shows the median value (horizontal line in the box). The box and whisker represent 25th to 75th, and 10th to 90th interquartile range, respectively. P values were determined by the Dunn’s post-test after Kruskal-Wallis test for comparison of three groups. (* p < 0.05 and ** p < 0.01). (TIF) [file pone.0200564.s005.tif]

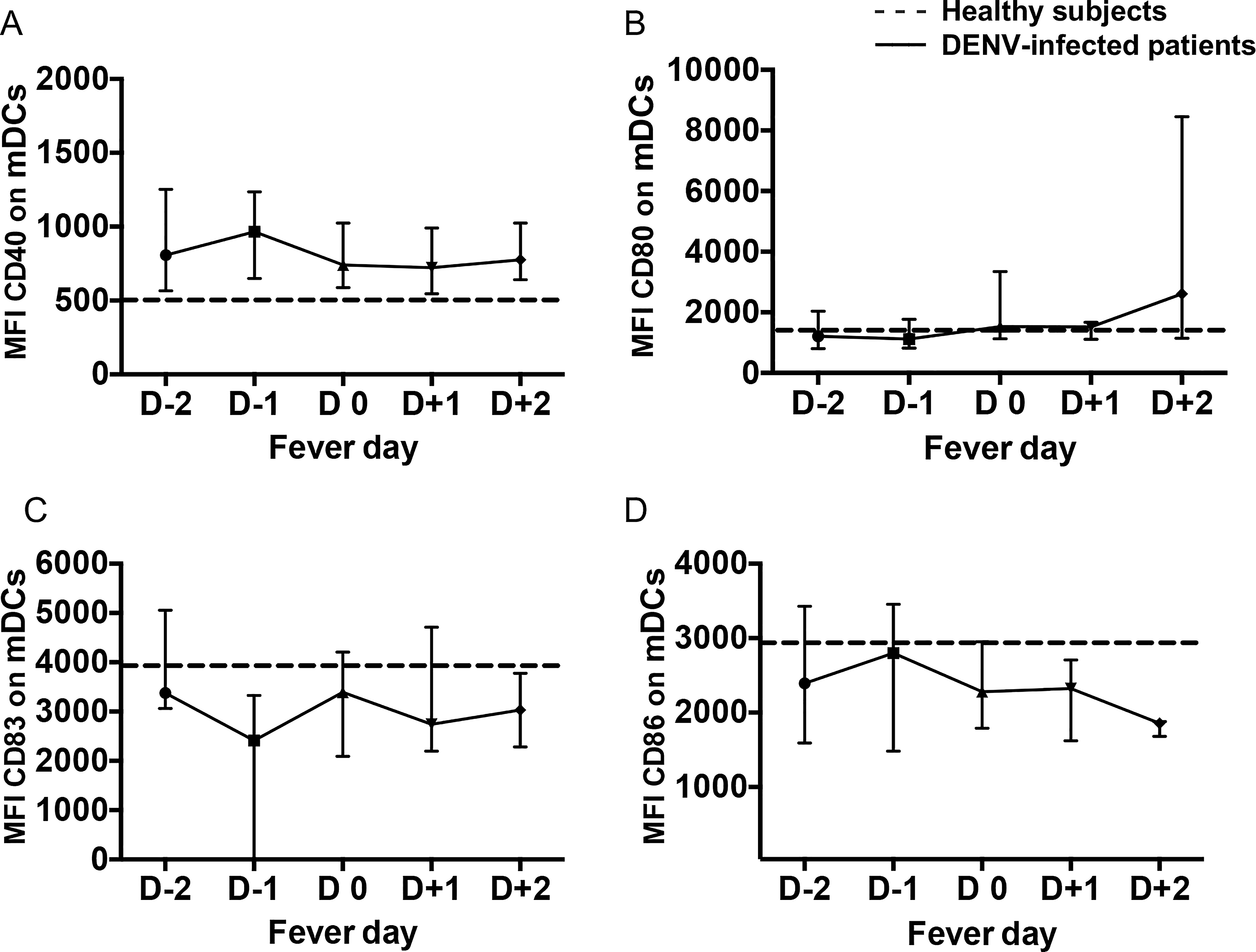

Supplement: S6 Fig — The MFI of CD40 (A), CD80 (B), CD83 (C) and CD86 (D) -expressing mDCs were determined at different days of fever ranging from febrile day-2 (D-2) and day-1 (D-1) to defervescence day 0 (D0) and day+1 (D+1) and to afebrile day+2 (D+2). Data are median and interquartile range. P values were determined by the Dunn’s post-test after Kruskal-Wallis test for comparison of three groups. The median of MFI on samples from healthy subjects are denoted by a dashed line. (TIF) [file pone.0200564.s006.tif]

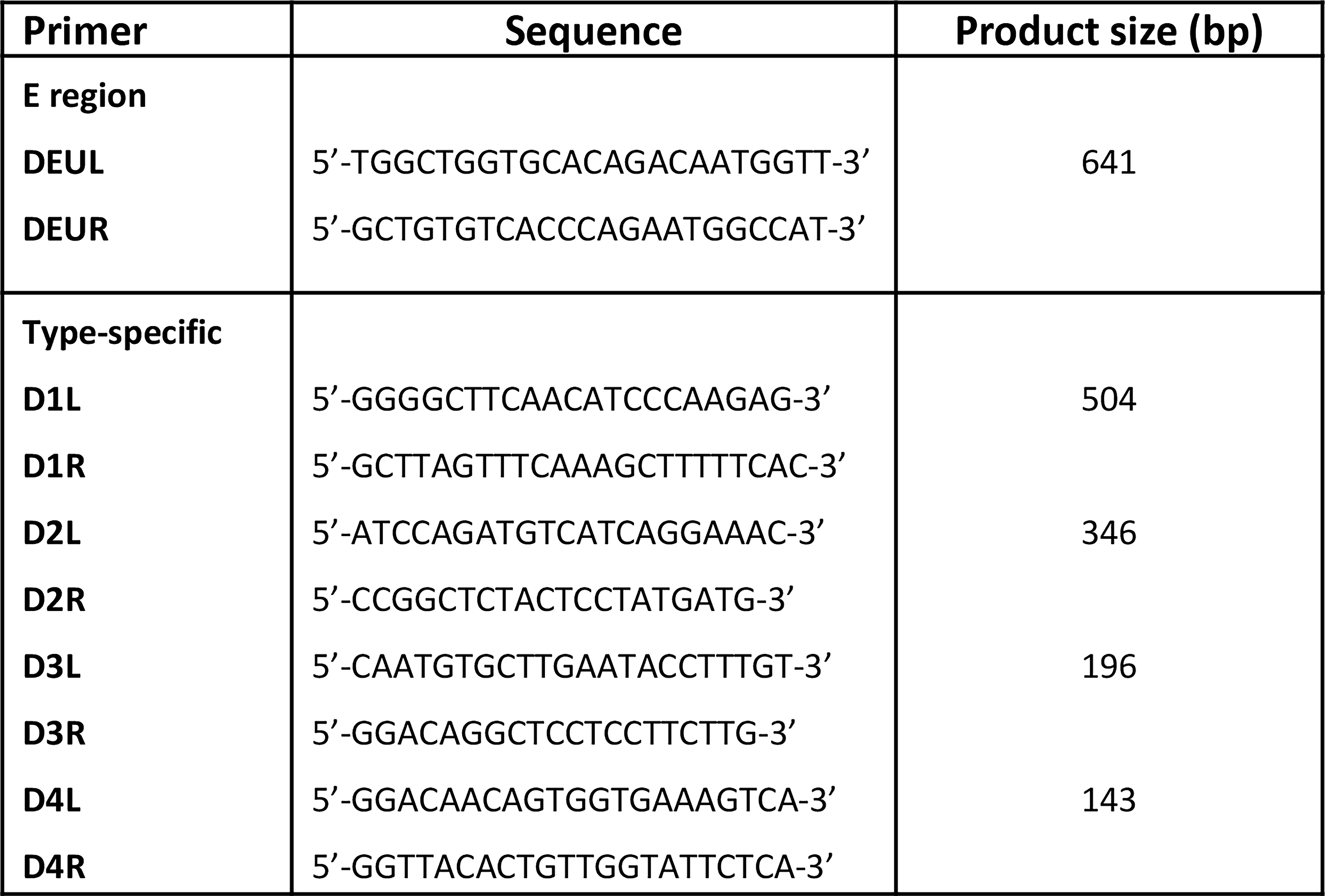

Supplement: S1 Table — (TIF) [file pone.0200564.s007.tif]

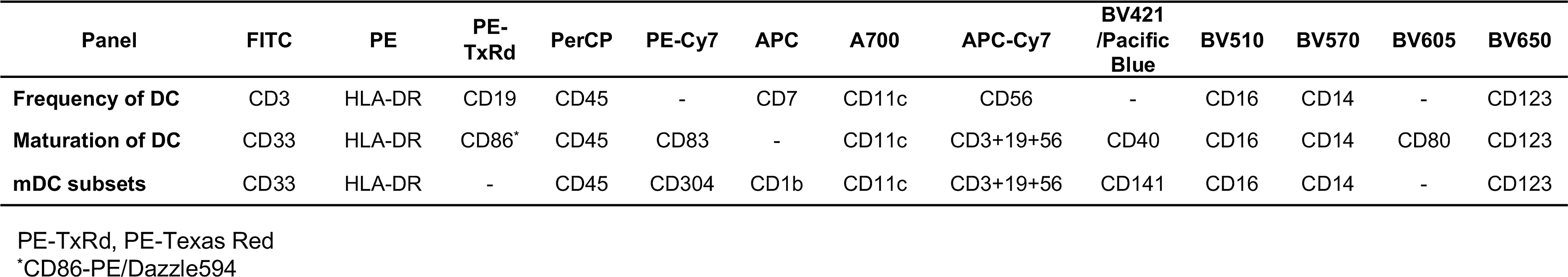

Supplement: S2 Table — (TIF) [file pone.0200564.s008.tif]
